# ﻿Taxonomic revision of Polygalaceae (Fabales) in Taiwan

**DOI:** 10.3897/phytokeys.262.162234

**Published:** 2025-09-01

**Authors:** Jo-Yu Wu, Hsy-Yu Tzeng, T. Y. Aleck Yang

**Affiliations:** 1 National Chung-Hsing University, Taichung, Taiwan National Chung-Hsing University Taichung Taiwan; 2 National Museum of Natural Science, Taichung, Taiwan National Museum of Natural Science Taichung Taiwan

**Keywords:** *
Epirixanthes
*, *
Heterosamara
*, morphology, palynology, *
Polygala
*, *
Salomonia
*, *
Senega
*, taxonomy

## Abstract

Several morphological and palynological characteristics—including petiole length, number of anthers, calyx persistence, presence of petaloid sepals, morphology of keel appendages, fruits, seed appendages, and shapes and polarity of pollen—were observed in this study. Five genera and nine species of the family Polygalaceae are recognized in Taiwan, including one new combination, *Heterosamara arcuata* (Hayata) J.Y. Wu & T.Y.A. Yang, **comb. nov.** Keys to the genera and species, taxonomic descriptions, tables summarizing characteristics, and photographs of each taxon are also provided.

## ﻿Introduction

Polygalaceae (order Fabales; The Angiosperm Phylogeny Group 2016) is a family with a broad global distribution, found in tropical, subtropical, and temperate regions. This family comprises 29 genera and approximately 1,200 species and can be divided into two subfamilies (Pastore et al. 2019; Pastore et al. 2023). Within the subfamily Polygaloideae, there are three tribes: Carpolobieae B. Eriksen, Diclidanthereae Reveal, and Polygaleae Fr., while the subfamily Xanthophylloideae contains one tribe, Xanthophylleae Baill. Species of Polygalaceae have long been recognized for their medicinal, ethnobotanical, and other significance (Eriksen and Persson 2007; Mongalo et al. 2015; Lacaille-Dubois et al. 2020). Despite their practical importance, the taxonomic classification of many Polygalaceae species remains unresolved. Given their significant medicinal and potential economic value, it is especially important to have a comprehensive understanding of the floras in various regions. Accurate classification and identification of these species rely on careful examination of their morphological characteristics, which are crucial for distinguishing and correctly classifying them. Polygalaceae classification is characterized by floral features such as keeled flowers, stamen number, and fruit type.

In recent years, molecular phylogenetic studies have emerged as a powerful and effective approach to addressing long-standing issues in plant taxonomy. This is particularly evident in Polygalaceae for delimiting the genus *Polygala* s.l. and recognizing the major evolutionary lineages within the tribe Polygaleae. For example, multiple phylogenetic analyses have demonstrated that *Polygala* s.l. does not constitute a monophyletic group (Abbott 2009; Pastore et al. 2017). Abbott (2009) proposed that *Polygala* s.l. comprises two stable and geographically distinct clades: one from the New World (NWC) and the other from the Old World (OWC). Subsequently, Pastore et al. (2023) formally recognized the New World clade as a separate genus, *Senega* Spach, based on a combination of molecular and morphological evidence, confirming that *Polygala* s.str. is primarily distributed in the Old World.

In addition to this significant taxonomic revision, recent phylogenetic studies have identified several well-supported clades within the tribe Polygaleae. A prominent example is the SECHP clade, which includes the genera *Salomonia* Lour., *Epirixanthes* Blume, Polygala subg. Chodatia Paiva, *Heterosamara* Kuntze, and *Polygaloides* Haller (Pastore et al. 2019; Pastore et al. 2023). Molecular data strongly support the SECHP clade as a monophyletic lineage, providing a clearer understanding of the evolutionary relationships within the tribe. Within the SECHP clade, *Salomonia* and *Epirixanthes* collectively form a distinct evolutionary branch as the sister group to the clade that comprises Polygala subg. Chodatia, *Heterosamara*, and *Polygaloides* (Pastore et al. 2019). However, the taxonomic delimitation and interrelationships between Polygala subg. Chodatia, *Heterosamara*, and *Polygaloides* remain unresolved. Overall, these findings have significantly refined our understanding of the taxonomic framework of the Polygalaceae.

Currently, three genera of Polygaleae have been recorded in Taiwan—*Epirixanthes* Blume, *Polygala* L., and *Salomonia* Lour.—comprising a total of nine species (Huang 1993, 2003; Yang and Chen 2013; Chung 2017). During our review of *Polygala* in Taiwan, we observed that the taxonomic information on Polygalaceae appears to be incomplete, and there may be a delay in incorporating the latest classification results from international molecular phylogenetic studies. For example, the reassignment of *P. tatarinowii* to *Heterosamara* as *H. tatarinowii* (Regel) Paiva was proposed by Paiva (1998), Banks et al. (2008), and Pastore et al. (2019). Additionally, *P. paniculata* L. was recently transferred to the genus *Senega* Spach by Pastore et al. (2023). However, these taxonomic changes have yet to be fully integrated into the current classification framework used in Taiwan, where more traditional systems remain prevalent and the incorporation of recent international findings in the family Polygalaceae is still limited.

Given these discrepancies, there is an urgent need to re-evaluate the current taxonomic treatments of Polygalaceae in Taiwan. This study conducts a comprehensive reassessment of Taiwanese species based on detailed morphological investigations, which include observations of freshly collected field material, herbarium specimens, and pollen morphology analyzed through scanning electron microscopy. Although molecular data are not included in the present analysis, recent phylogenetic findings are considered to ensure that the proposed taxonomic revisions align with contemporary global systematic frameworks. By integrating traditional morphological approaches with insights from recent molecular studies, this work aims to clarify taxonomic boundaries, improve consistency with international classifications, and enhance our understanding of local species diversity. Additionally, it contributes to the conservation, sustainable use, and further study of these taxa, which have medicinal and economic significance.

## ﻿Materials and methods

We observed a total of 202 specimens, either on loan or as specimen photographs, from the following herbaria: BM, K, MA, PPI, TAI, TAIF, TCF, and TNM. The morphological characteristics of leaves, flowers, fruits, and seeds were examined from fresh specimens and dried samples using the TORI FOCUS, developed by the Taiwan Ocean Research Institute (TORI), along with the VHX-970F digital microscope and the FUJIFILM X-T2 camera. Pollen grains were acetolyzed according to the method proposed by Erdtman (1960) and observed using the HITACHI SU-1510 scanning electron microscope (SEM). The fresh plants collected during our study have been preserved and are now housed in the National Museum of Natural Science (NMNS). Voucher specimens have been deposited at the TNM herbarium (National Museum of Natural Science, NMNS) and the TCF herbarium (Department of Forestry, National Chung Hsing University, NCHU). For each species, two specimens from each county are cited in the text, with additional specimens listed in Appendix 1. All specimens examined for morphological measurements and figures are listed in Appendix 2 with complete collection data, including collector, collection number, date, and herbarium codes.

## ﻿Results

### ﻿Morphological characteristics

All nine species were examined based on fresh materials and herbarium specimens (Table 1).

**Table 1.** Summary of characteristics of Polygalaceae in Taiwan.

Taxon		Epirixanthes elongata	Heterosamara tatarinowii	Polygala arcuata	P. arvensis	Polygala chinensis	P. japonica	P. polifolia	Salomonia ciliata	Senega paniculata
life-form		erect mycoheterotrophic herbs	erect herbs	erect subshrubs	erect or ascending herbs	erect herbs	erect or ascending herbs	erect or ascending herbs	erect herbs	erect herbs
leaves	shape	ovate	obovate to spatulate	lanceolate to narrowly elliptic	narrowly elliptic to oblanceolate	elliptic to narrowly elliptic	lanceolate, ovate, elliptic to narrowly elliptic	ovate to elliptic	lanceolate to ovate	linear
	petiole length (mm)	sessile	3.2–7.0	4.0–25.0	1.5–1.8	0.5–2.3	0.5–1.0	0.4–0.8	0.2–0.5	0.5–1.2
	texture	scarious	herbaceous	membranous	chartaceous	chartaceous	chartaceous	chartaceous	chartaceous	herbaceous
flowers	sepals	non-petaloid; sepals flat, persistent	two lateral ones petaloid; sepals revolute, caducous, glabrous	two lateral ones petaloid; sepals revolute, caducous, glabrous	two lateral ones petaloid; sepals flat, persistent	two lateral ones petaloid; sepals flat, persistent	two lateral ones petaloid; sepals flat, persistent	two lateral ones petaloid; sepals flat, persistent	non-petaloid; sepals flat, persistent	two lateral ones petaloid; sepals flat, persistent
	petals	white	purple	purple	orange-yellow	white with magenta spots	purple to violet, few white	violet	purple	white
	keel appendages	absent	absent	yellow, crest-like, unipinnate	yellow, brush-like, bipinnate to tripinnate	pale red, deer horn-like, bipinnate to tripinnate	violet, fringe-like, bipinnate to tripinnate, or more	violet, brush-like, unipinnate to bipinnate	absent	white, finger-like, unipinnate
	number of anthers	5	8	8	8	8	8	8	4	8
	stigmas	unitary	bifid	bifid	unitary	unitary	bifid	bifid slightly	unitary	bifid
fruits	margin	entire	entire	entire	entire, with long cilia	entire, with long cilia	entire	entire, with long cilia	with teeth and spines	entire
	surface	hairy	glabrous	glabrous	hairy	hairy	hairy	hairy	hairy	hairy
seeds	aril	flaky, membranous	3-lobed, terminal membranous	4-lobed, flaky, membranous	simple, tiny	3-lobed, terminal membranous	3-lobed, hypertrophic	3-lobed, terminal membranous	arils subtle, membranous	2-lobed, terminal membranous, extend
	surface	glabrous	sparse hairs	sparse hairs	densely hairs	densely hairs	sparse hairs	densely hairs	glabrous	sparse hairs

#### ﻿Leaves

Leaf morphology across Polygalaceae exhibits considerable diversity. Leaf texture is a particularly informative character, ranging from scarious forms in *Epirixanthes* to succulent, membranous, and herbaceous adaptations in *Heterosamara tatarinowii*, *Polygala arcuata*, and *P. paniculata*, with the remaining species displaying chartaceous leaves. Petiole length variation shows taxonomic significance—*H. tatarinowii* and *P. arcuata* exhibit notably longer petioles (>3 mm) compared to other taxa. These leaf variations likely represent multiple independent adaptations to different water regimes and light conditions across the diverse habitats occupied by the family.

#### ﻿Flowers

Flowers of this family typically possess five sepals and three petals that are connate at the base, with four to eight anthers and variable keel appendages. Key diagnostic characters at the genus level include anther number, presence or absence of petaloid sepals, and sepal persistence. Keel appendage morphology exhibits considerable diversity across the family in Taiwan, ranging from complete absence in *H. tatarinowii* to highly complex multipinnate structures in other taxa.

#### ﻿Fruits

Fruits of Polygalaceae are typically capsules, either dehiscent or indehiscent. Capsules of *Epirixanthes* are spheroidal and indehiscent at maturity, while the other genera produce flat and dehiscent fruits in Taiwan. Key diagnostic features include fruit dehiscence, surface pubescence, and margin ornamentation. Fruit margins exhibit variation across the family in Taiwan, ranging from entire margins in most species to specialized structures such as spine-like teeth in *Salomonia ciliata* and long cilia in several *Polygala* species.

#### ﻿Seeds

Seeds of Polygalaceae in Taiwan are generally ovoid to ellipsoid with membranous arils attached to the caruncles. Arils and caruncles provide both dispersal and taxonomic significance, and aril morphology ranges from subtle or absent structures to multi-lobed forms. Seed surface pubescence also shows diagnostic variation, from glabrous to densely hairy surfaces among Taiwanese taxa.

### ﻿Palynological characteristics

**Figure 1.**
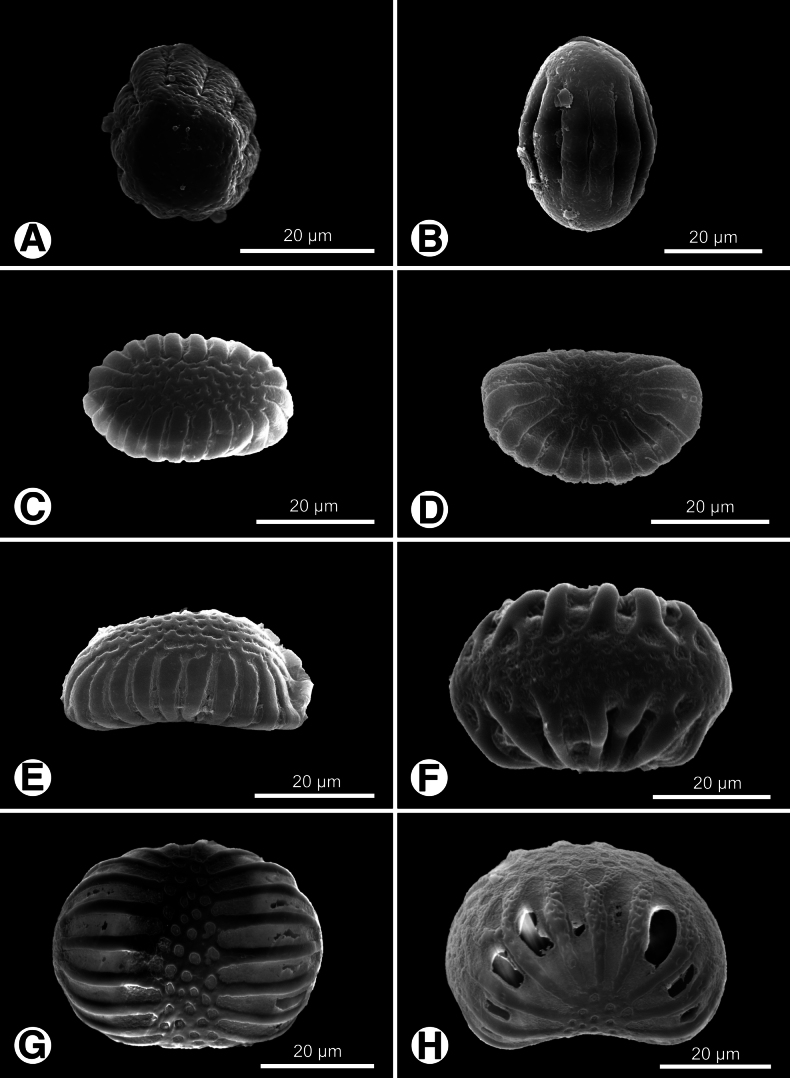
Pollen grains of *Epirixanthes*, *Heterosamara*, and *Polygala* in Taiwan. **A, B.***E. elongata*; **C–E***H. tatarinowii*; **F–H.***P. arcuata*. **A, C, D, F, G.** Polar view; **B, E, H.** Equatorial view.

**Figure 2.**
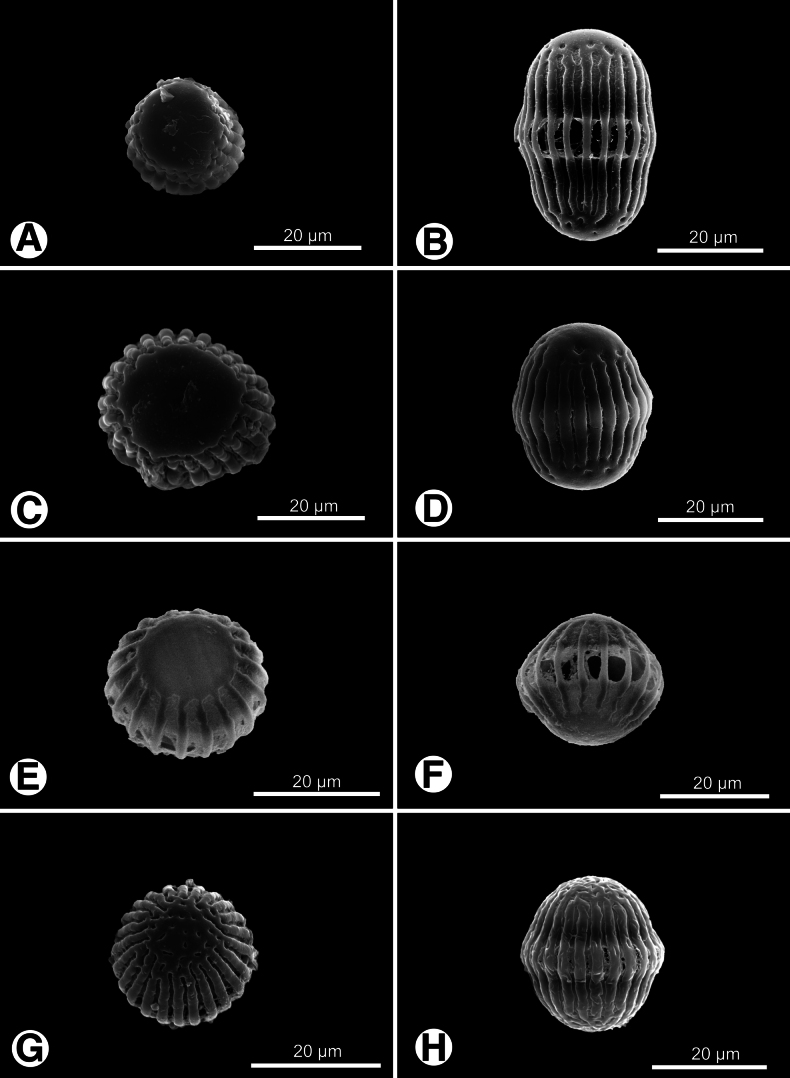
Pollen grains of *Polygala* in Taiwan. **A, B.***P. arvensis*; **C, D.***P. chinensis*; **E, F.***P. japonica*; **G, H.***P. polifolia*. **A, C, E, G.** Polar view; **B, D, F, H.** Equatorial view.

**Figure 3.**
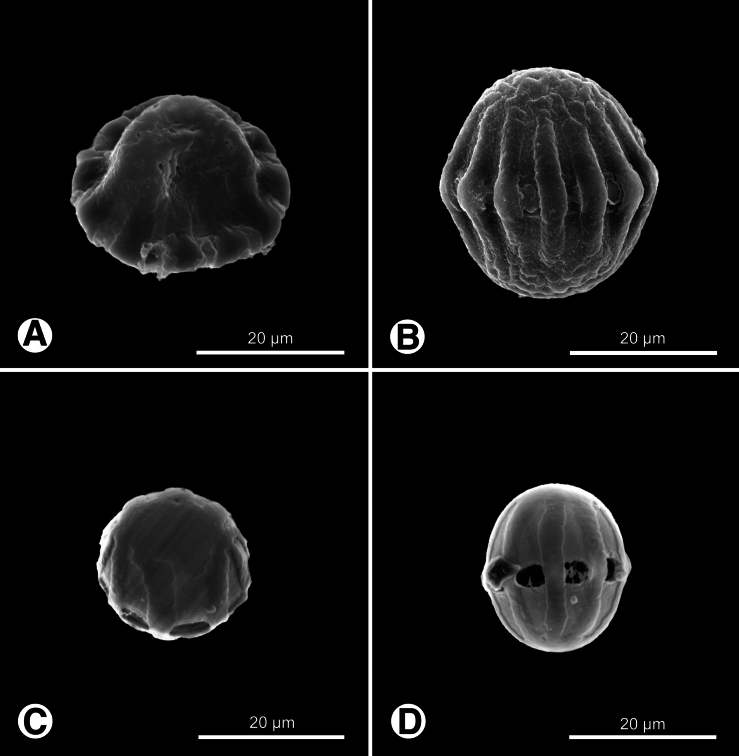
Pollen grains of *Salomonia* and *Senega* in Taiwan. **A, B.***Salomonia ciliata*; **C, D.***Senega paniculata*. **A, C.** Polar view; **B, D.** Equatorial view.

Pollen grains of Polygalaceae in Taiwan are polycolporate, with aperture numbers ranging from eight to 26. Key diagnostic features include polarity, grain shape, and exine ornamentation patterns. Most genera exhibit isopolar pollen with spheroidal to prolate shapes in equatorial view (Figs 1A, B, 2A–H, 3A–D), while *H. tatarinowii* and *P. arcuata* show heteropolar, kidney-shaped grains (Fig. 1C–H). Exine ornamentation provides additional taxonomic significance, ranging from rugulate-perforate to rugulate-foveolate and psilate patterns among the examined taxa. Additional details can be found in Table 2.

**Table 2.** Summary of pollen characteristics of Polygalaceae in Taiwan.

Taxon	size (P × E, µm)	P/E	number of apertures	isopolar or heteropolar	exine patterns
*Epirixanthes elongata*	34.7–41.6 × 23.3–31.9	1.20–1.52	10–12	isopolar	regulate, perforate
*Heterosamara tatarinowii*	14.5–22.6 × 31.4–41.1	0.41–0.69	20–24	heteropolar	foveolate, reticulate
*Polygala arcuata*	23.9–34.2 × 42.3–57.1	0.47–0.76	20–24	heteropolar	regulate, foveolate with few circular lumina
*P. arvensis*	27.9–39.4 × 20.4–26.3	1.19–1.55	21–23	isopolar	psilate or foveolate
*P. chinensis*	27.6–34.5 × 23.2–33.6	1.00–1.30	21–23	isopolar	psilate, sometimes foveolate
*P. japonica*	19.0–29.2 × 17.6–24.1	0.71–1.09	14–19	isopolar	psilate
*P. polifolia*	20.2–26.6 × 23.1–26.4	0.90–1.14	22–26	isopolar	foveolate, reticulate
*Salomonia ciliata*	27.1–28.4 × 27.5–33.3	0.91–0.99	10–14	isopolar	foveolate, reticulate
*Senega paniculata*	20.5–22.6 × 23.8–26.2	0.94–1.27	8–12	isopolar	psilate

## ﻿Discussion

Five genera can be distinguished by several morphological characteristics described in the preceding section. Based on the larger, softer, and membranous leaves with petioles measuring 4–25 mm in length; revolute, caducous, and glabrous sepals; bilobed keel appendages; glabrous fruits; and heteropolar, kidney-shaped pollen, we have separated *Polygala arcuata* from other species within the genus *Polygala*. Consequently, we have transferred *P. arcuata* to the genus *Heterosamara* as a new combination—*H. arcuata* (Hayata) J.Y. Wu & T.Y.A. Yang.

### ﻿Taxonomic treatment

#### ﻿ Polygalaceae

Taxon classificationPlantaeFabalesPolygalaceae

Hoffmanns. & Link, Fl. Portug. 1: 62. 1809 nom. cons.

51A1DCA2-B3E5-5E0F-9DC3-9E365A60A644

##### Description.

Subshrubs or herbs, erect or ascending, rarely achlorophyllous. Leaves simple, alternate, entire, exstipulate. Racemes terminal or axillary. Flowers bisexual, bilaterally symmetrical. Sepals five, imbricate, usually petaloid. Petals three, connate up to half their length, the abaxial one developed into a keel. Androecium monadelphous, filaments connate and fused with keel, anthers 4–8, dehiscent by apical pores. Ovaries 2-loculed, with one ovule in each locule, styles simple, stigmas 1–2. Capsules glabrous or hairy, dehiscent or indehiscent. Seeds two, glabrous or hairy, with arils. Pollens polycolporate, isopolar or heteropolar, spheroidal, prolate to oblate or kidney-shaped, apertures 8–26.

### ﻿Key to genera of Polygalaceae in Taiwan

**Table:** 

1	Herbs achlorophyllous	***Epirixanthes* (1)**
–	Herbs or subshrubs with green leaves	**2**
2	Calyces non-petaloid; anthers 4; fruit margin with teeth and spines	***Salomonia* (2)**
–	Calyces petaloid; anthers 8; fruit margin entire	**3**
3	Sepals revolute, caducous; petioles more than 3 mm long	***Heterosamara* (3)**
–	Sepals flat, persistent; petioles less than or equal to 3 mm long	**4**
4	Keel appendage fimbriated, broadly semicircular, unipennate	***Senega* (4)**
–	Keel appendage brush-like, deer-horn-like, bipinnate, or tripinnate	***Polygala* (5)**

#### ﻿ Epirixanthes

Taxon classificationPlantaeFabalesPolygalaceae

Blume, Catalogus: 25. 1823.

3C764034-241C-5B96-80B1-ED8FC3FEB780

##### Description.

Herbs erect, achlorophyllous. Leaves bract-like, widely ovate. Racemes terminal. Flowers dense. Sepals five, non-petaloid, flat, persistent after flowering. Petals three, connate up to half, keel without appendage. Filaments connate, anthers five. Styles straight or slightly curved, stigmas simple, capitate. Capsules glabrous, indehiscent. Seeds two, glabrous, with arils. Pollens isopolar, subprolate to prolate, radially symmetrical.

##### Distribution.

*Epirixanthes* contains 7 achlorophyllous species distributed from subtropical to tropical Asia and the South Pacific, and 1 species in Taiwan.

#### ﻿1. Epirixanthes elongata

Taxon classificationPlantaeFabalesPolygalaceae

Blume, Cat. Gew. Buitenzorg (Blume) 25. 1823; Hsieh et al. in Taiwania 40(4): 381–384. 1995.

6DE9C782-8ED1-5765-8238-2AA709E627AA

**Figure 4.**
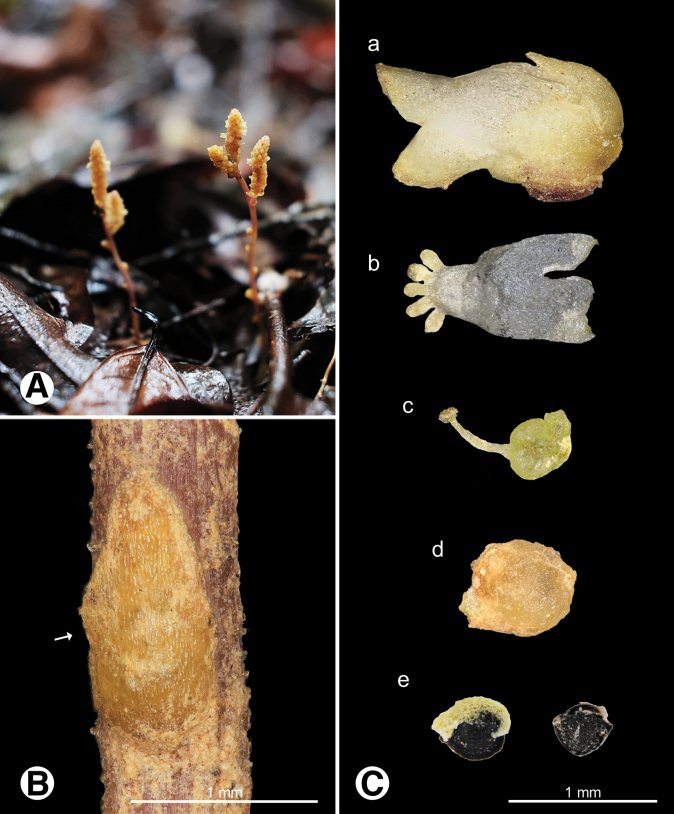
Photo of *Epirixanthes elongata*. **A.** Plants in field; **B.** Leaf (arrow indicates bract-like leaf on the stem); **C.** Reproductive organs a flower b androecium c gynoecium d fruit e seeds.

Figs 1A, B, 4 Common name. 寄生鱗葉草

##### Type specimen.

Indonesia, Java, *s.coll. s.n.*, s.d., (holotype: L photo!).

##### Description.

Herbs erect, 10–25 cm tall. Leaves simple, alternate, bract-like, ovate, sessile. Racemes terminal. Flowers white. Sepals five, persistent after flowering. Petals three, white, connate. Filaments connate, anthers five. Capsules glabrous, indehiscent. Seeds glabrous, with membranous arils.

Pollens isopolar, apertures 10–12. Subprolate to prolate, exine regulate and perforate. P/E ca. 1.20–1.52.

##### Distribution.

India, Bhutan, Bangladesh, Myanmar, Thailand, Laos, Vietnam, Malaysia, Brunei Darussalam, Indonesia, southern China, southern Taiwan, and the Solomon Islands (Van der Meijden 1988; Hsieh et al. 1995; Chen et al. 2008; Pendry 2010; Tsukaya et al. 2016; Dančák et al. 2017). Under forest.

##### Specimens examined.

**India. Assam State**: Dima Hasao dist., Haflong, N. Cadrar, *W.G. Craib s.n.*, 24 Aug. 1908 (K). **Myanmar.** Dawei Dist., Area within a radius of 12 miles from Paungdaw, half a mile east of Paungdaw Power Station on the east bank of the Paungdaw River, *J. Keenan et al. 1527*, Sep. 1961 (K). **Malaysia. Perak State**: Gunong Kerbau, *H.C. Robinson s.n.*, s.d. (K). **Sabah State**: Imbak Canyon, *R.P. Clark et al. 22*, 16 May 2004 (K). **Thailand.** Kaosuarj, N. Sritermarut, *A.F.G. Kerr 15434*, 28 Apr. 1928 (K). **Indonesia**, Java Island, *s.coll. s.n.*, 1863 (K). **Taiwan. Pingtung Co.**: Mutan Township, *C.M. Wang et al. 16992*, s.d. (TNM); Shihzih Township, Mt. Maotzushan, *J.Y. Wu et al. 627*, 6 Sep. 2024 (TCF).

#### ﻿ Heterosamara

Taxon classificationPlantaeFabalesPolygalaceae

Kuntze, Revis. Gen. Pl. 1: 47. 1891.

ADEC28F9-74E5-53CA-AD28-37E8744DB2F0

##### Description.

Subshrubs or herbs, perennial or annual, glabrous or with short hairs. Leaves simple, alternate, obviously petiolate, ovate to lanceolate, fleshy to membranous, lateral veins easily visible. Racemes terminal or axillary. Flowers purple, dense. Sepals five, of three small, outer, and two large, inner. Sepals petaloid, glabrous, revolute, caducous. Petals three, connate at base up to half, keel appendages bilobed or absent. Filaments connate at middle and fused with keel, anthers eight. Styles slightly curved, stigmas bifid. Capsules glabrous, entire, green at maturity. Seeds two, glabrous to densely hairy, ellipsoidal, with arils. Pollens heteropolar, kidney-shaped, bilaterally symmetrical. Exine patterns regulate and foveolate.

##### Distribution.

*Heterosamara* contains 18 species distributed in tropical to subtropical Africa and Asia, two species found in Taiwan.

### ﻿Key to the species of *Heterosamara* Kuntze in Taiwan

**Table:** 

1	Subshrubs; leaves lanceolate; keel appendages yellow, crest-like	***H. arcuata* (1)**
–	Herbs; leaves obovate; keel appendages absent	***H. tatarinowii* (2)**

#### ﻿1. Heterosamara arcuata

Taxon classificationPlantaeFabalesPolygalaceae

(Hayata) J.Y. Wu & T.Y.A. Yang comb. nov.

BAA2F83F-6EF8-5D19-96CD-AEBB3368D683

urn:lsid:ipni.org:names:77368454-1

**Figure 5.**
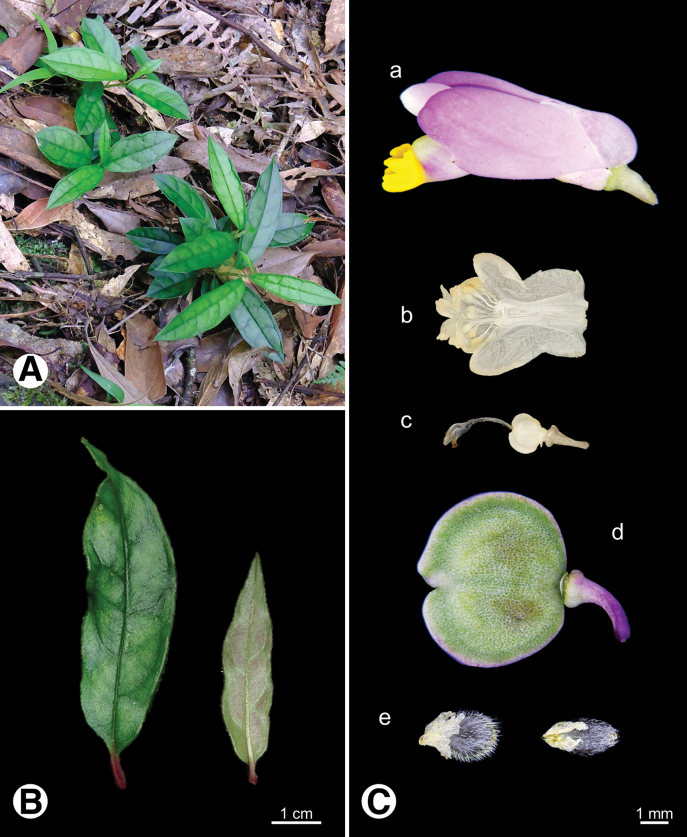
Photo of *Heterosamara arcuata***A.** Plants in field; **B.** Leaves; **C.** Reproductive organs a flower b androecium c gynoecium d fruit e seeds.

Figs 1F–H, 5 Common name. 巨葉花異翅果 (新擬)

Polygala crassiuscula Hayata, Icon. Pl. Formosan. 3: 32. 1913. Type specimen: TAIWAN. Chiayi, Alisan, *S. Sasaki s.n.*, Jan. 1911(TI).

##### Basionym.

*Polygala arcuata* Hayata, J. Coll. Sci. Imp. Univ. Tokyo 25(19): 54. 1908. Type specimen: TAIWAN. Taichung, Kashigatani, *G. Nakahara s.n.*, Feb. 1907 (isotype: TAIF!).

##### Type specimen.

Taiwan, Taichung, Kashigatani, *G. Nakahara s.n.*, Feb. 1907 (isotype: TAIF!).

##### Description.

Subshrubs, up to 60 cm tall. Leaves simple, alternate, petioles 4–25 mm long, blades 3–10 cm long, 1–3 cm wide, elliptic to lanceolate, glabrous, fleshy, pale green; midrib and lateral vein dark green, obvious. Racemes 2.5–7 cm long, terminal or axillary. Flowers rarely purple or white. Sepals purple, petaloid, revolute, caducous. Petals three, connate, upper 1/3 free, keel pinkish purple, appendages crest-like, yellow. Filaments connate at base, free at upper 1/3 portion, anthers eight. Ovaries bilocular, ovule one per locule, stigmas bifid, sterile one glabrous. Capsules flat, green at maturity. Seeds ovate, with sparse, short hairs, arils 4-lobed, flaky, membranous.

Pollens heteropolar, apertures 20–24. Kidney-shaped and prolate in equatorial view. Exine patterns regulate, foveolate, polar view with some circular lumina. P/E ca. 0.47–0.76.

##### Distribution.

Endemic to Taiwan (Huang 1977, 1993; Yang and Chen 2013), distributed in forest edges at the altitude of 1,200–3,000 m in central and southern Taiwan.

##### Specimens examined.

**Taiwan. Taichung City**: Hoping Dist., Mt. Pahsienshan, *J.Y. Wu et al. 680*, 29 Nov. 2024 (TCF); Mt. Paimaoshan, *J.Y. Wu 101*, 19 Feb. 2022 (TNM). **Nantou Co.**: Renai Township, Mt. Hsiaochushan, *F.C. Kuo & S.Y. Lu 3*, 10 Mar. 2007 (TAIF), *I.C. Kao 26*, 27 Dec. 2009 (TAIF). **Pingtung Co.**: Chunrih Township, Chinshuiying, *J.Y. Wu et al. 676*, 24 Nov. 2024 (TCF); Taiwu Township, Mt. Peitawushan, *J.Y. Shen et al. 20220816*, 16 Aug. 2022 (TNM). **Taitung Co.**: Jinfong Township, Mt. Talilishan, *T.C. Hsu 6911*, 1 Mar. 2014 (TAIF).

#### ﻿2. Heterosamara tatarinowii

Taxon classificationPlantaeFabalesPolygalaceae

(Regel) Paiva, Fontqueria 50: 130. 1998.

77634049-70B5-5AE3-93C1-3B805A8855F2

**Figure 6.**
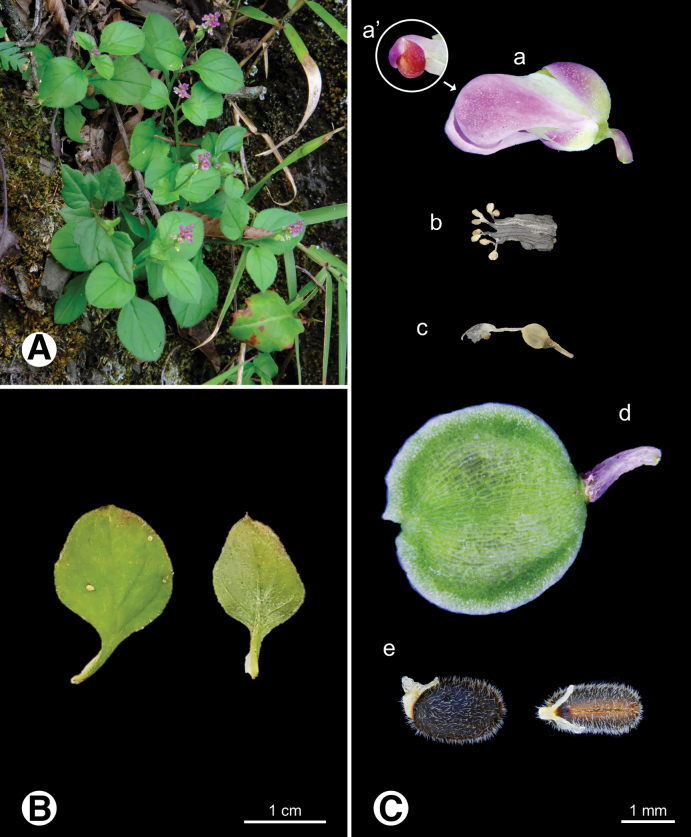
Photo of *Heterosamara tatarinowii***A.** Plants in field; **B.** Leaves; **C.** Reproductive organs a flower a’ keel apical, appendages absent b androecium c gynoecium d fruit e seeds.

Figs 1C–E, 6 Common name. 小扁豆

##### Basionym.

*Polygala tatarinowii* Regel in Bull. Soc. Nat. Mosc. 34 2(4): 523. 1861; Huang in Fl. Taiwan 3 (1^st^ edn.): 560, *pl. 727*. 1977. Type specimen: CHINA. near Beijing (Peking), *A.A. Tatarinow 957* (holotype: LE!).

##### Type specimen.

China, near Beijing (Peking), *A.A. Tatarinow 957* (holotype: LE!).

##### Description.

Herbs erect, up to 15 cm tall. Leaves simple, alternate, petioles 3.2–7 mm long, blades 0.7–4.1 cm long, 0.3–2.2 cm wide, ovate, membranous, with short hairs. Racemes 2.3–5 cm long, terminal or axillary. Flowers purple. Sepals five, purple, petaloid, revolute, caducous. Petals three, keel yellow or red, appendages absent. Filaments connate at base, free at upper 1/3 portion, anthers eight. Ovaries bilocular, ovule one per locule, stigmas bifid, sterile one palmate, glabrous. Capsules flat, yellowish green at maturity. Seeds ovate, with dense, short hairs, arils 3-lobed, membranous.

Pollens heteropolar, apertures 20–24. Kidney-shaped, equatorial view prolate. Exine foveolate and reticulate. P/E ca. 0.41–0.69.

##### Distribution.

Pakistan, India, Bhutan, Myanmar, Vietnam, Papua New Guinea, the Philippines, China, Japan, Korea, and Taiwan (Huang 1977, 1993; Yang and Chen 2013). Mainly distributed in forest edges and roadsides at low to middle altitudes in central, southern, and eastern Taiwan.

##### Specimens examined.

**India.** Himachal Pradesh State, Chil Forest above Waterfalls Simla, *J.S. Gamble 5091A*, 16 Sep. (K). Lachooni, *J.D.H. s.n.*, 15 Aug. (K). **Bhutan.** Thimphu Dist., Dechhenphu N of Thimphu, *I.W.J. Sinclair & D.G. Long 4817*, 5 Sep. 1984 (K). **China.** Yunnan Dist., *J. Cavalerie 4394*, 1917 (K). **Japan.** Hondo, Nippara in Musashi, *M. Togasi 1223*, 14 Sep. 1955 (K). Mrioka, *P. Faurie 618[3*], 28 Aug. 1890 (K). **Taiwan. Hsinchu Co.**: Jianshih Township, Taikang, *P.F. Lu 25953*, 10 Aug. 2013 (TAIF). **Nantou Co.**: Renai Township, *J.Y. Wu 212*, 10 Aug. 2022 (TNM); Tunyuan, *T.Y.A. Yang et al. 19771*, 17 Sep. 2007 (TNM). **Chiayi Co.**: Alishan Township, Alishan Highway, *C.M. Wang et al. 17150*, 12 Sep. 2017 (TNM); Jhuci Township, Shihcho, *T.C. Hsu 4651*, 15 Sep. 2011 (TAIF). **Kaohsiung City**: Taoyuan Dist., Tianchi, *C.S. Kuoh 15022*, 14 Sep. 1999 (TNM); Bridge Likuangchiao, *T.Y.A. Yang et al. 7488*, 13 Sep. 1996 (TNM). **Ilan Co.**: Datong Township, Suchi-Nanshan, *C.M. Wang s.n.*, 6 Aug. 1992 (TNM). **Hualien Co.**: Shoufong Township, National Dong-Hwa University, *C.T. Chao Chao4750*, 18 Feb. 2019 (TAIF); Tienchih, *P.F. Lu 29811*, 21 Aug. 2016 (TAIF). **Taitung Co.**: Haiduan Township, Tienlung Historical Trail, *P.F. Lu 10563*, 16 Oct. 2005 (TAIF).

#### ﻿ Polygala

Taxon classificationPlantaeFabalesPolygalaceae

L., Sp. Pl.: 701. 1753.

C81A532F-0030-5CF9-A128-152047609ADD

##### Description.

Herbs, shrubs, or small trees. Leaves simple, alternate, estipulate, petiolate, entire, glabrous, or pilose. Racemes terminal or axillary. Flower purple, violet, white, or yellow. Sepals five, petaloid, flat, persistent, consisting of three small, outer, and two large, inner. Petals three, connate up to the half, keel appendages unipinnate to tripinnate. Filaments connate at half and up to upper, anthers eight. Ovaries 2-loculed, ovule one per locule, styles simple, curved sometimes, stigmas one, rarely two, gradually curved and thickened. Capsules pilose. Seeds two, glabrous, densely hairs, ellipsoidal, appendages present. Pollens isopolar, oblate, spheroidal, subprolate to prolate, radially symmetrical.

##### Distribution.

About 550 species worldwide, with four species found in Taiwan.

### ﻿Key to the species of *Polygala* L. in Taiwan

**Table:** 

1	Lateral veins of adaxial leaves clearly visible; seeds with hypertrophic arils	***P. japonica* (1)**
–	Lateral veins of adaxial leaves obscure; seeds with membranous arils or not	**2**
2	Leaves oblanceolate; flowers yellow	***P. arvensis* (2)**
–	Leaves elliptic or ovate; flowers white or violet	**3**
3	Keel appendages apex tapered; flowers white with magenta spots	***P. chinensis* (3)**
–	Keel appendages apex rounded; flowers violet	***P. polifolia* (4)**

#### ﻿1. Polygala arvensis

Taxon classificationPlantaeFabalesPolygalaceae

Willd., Sp. Pl., 3(2)(4th edn.): 876. 1802; Chung in Illustrated flora of Taiwan 4: 276. 2017.

1CB74701-331E-5F06-B4FE-3C4E4BDF7E78

**Figure 7.**
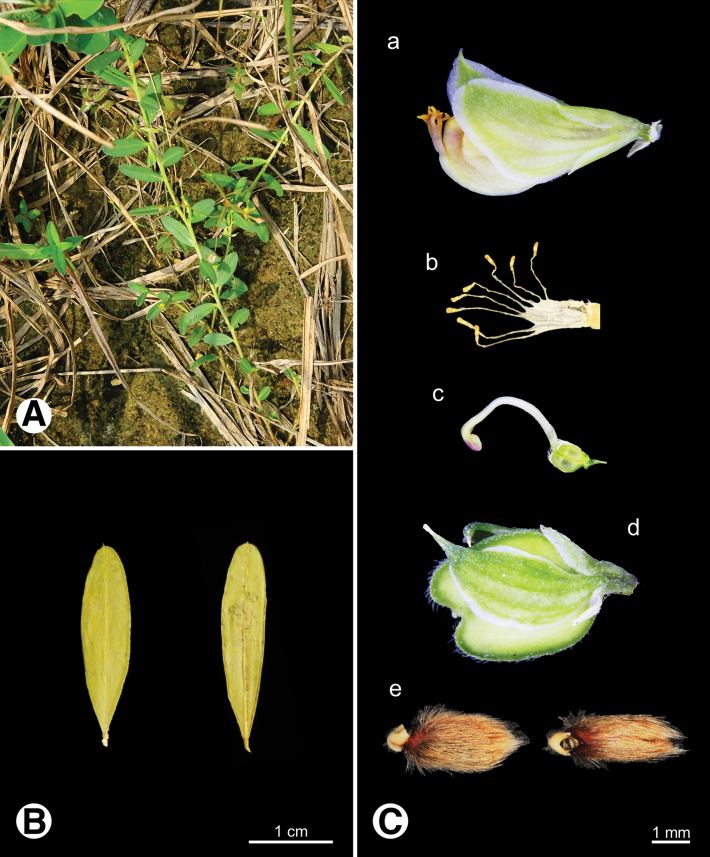
Photo of *Polygala arvensis***A.** Plant in field; **B.** Leaves; **C.** Reproductive organs a flower b androecium c gynoecium d fruit e seeds.

Figs 2A, B, 7 Common name. 小花遠志

##### Type specimen.

India, prope Madrastam, *s.coll. s.n.*, 25 Aug. 1794 (holotype: B photo!).

##### Description.

Herbs, erect or ascending. Leaves simple, alternate, narrowly elliptic to oblanceolate, petioles approximately 1.5–1.8 mm. Blades chartaceous. Racemes axillary. Flowers yellow. Sepals petaloid, flat, persistent. Petals three, keel appendages orange-yellow, brush-like. Filaments connate at the base, free at middle, anthers eight. Ovaries 2-loculed, ovule one per locule, styles simple, curved, stigmas simple. Capsules flat. Seeds ellipsoid, with dense long hairs, arils 2-lobed.

Pollens isopolar, apertures 21–23. Subprolate to prolate, exine psilate, sometimes foveolate. P/E ca. 1.19–1.55.

##### Distribution.

Pakistan, India, Malaysia, Vietnam, Taiwan, Nepal, and northern and western Australia (Bashyal et al. 2025; POWO 2025). In Taiwan, it is only found in open grasslands at low elevations in Tainan City.

##### Specimens examined.

**India.** Goalpara, *s.coll. 4172*, s.d. (K). Murree-Kohala Road, *R.R. Stewart 5068*, 30 Aug. 1919 (K). **Taiwan. Tainan City**: Guanmiao Dist., *T.H. Hsieh s.n.*, 11 Oct. 2020 (TNM); Xinhua Dist., Mujiashan Grasslands, *J.Y. Wu 63*, 2 Oct. 2021 (TNM).

#### ﻿2. Polygala chinensis

Taxon classificationPlantaeFabalesPolygalaceae

L., Sp. Pl.: 704. 1753; Yang & Chen in Taiwania 58(3): 156–162. 2013.

48790D39-96E9-5D3D-A56F-35704D2682EA

**Figure 8.**
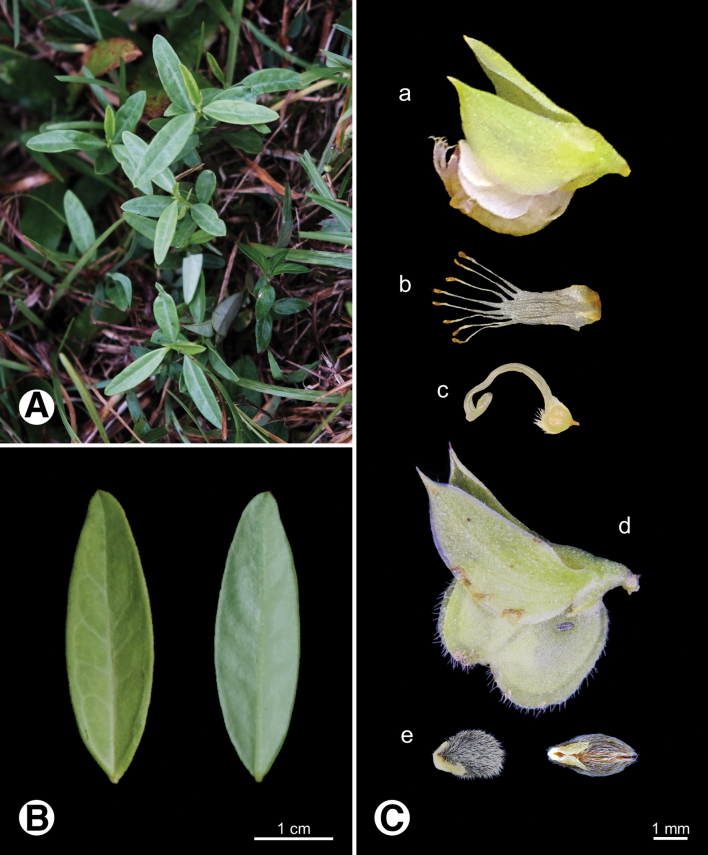
Photo of *Polygala chinensis***A.** Plants in field; **B.** Leaves; **C.** Reproductive organs a flower b androecium c gynoecium d fruit e seeds.

Figs 2C, D, 8 Common name. 華南遠志

Polygala glomerata Lour., Fl. Cochinch. 2: 426. 1790; Henry, List Pl. Formos. 18. 1896. Type specimen: CHINA. Guangdong (Canton), *J. de Louriero. s.n.*, s.d. (holotype: P!).

##### Type specimen.

India. *C. Linnaeus s.n.*, s.d. (lectotype: LINN!).

##### Description.

Herbs erect. Leaves simple, alternate, elliptic to narrowly elliptic, petioles approximately 0.5–2.3 mm. Blades chartaceous. Racemes axillary. Sepals petaloid, flat, persistent. Petals three, white, with magenta spots, keel appendages pale red, deer horn-like. Filaments connate at base, free at middle, anthers eight. Ovaries bilocular, ovules two, styles simple, curved, stigmas simple. Capsules flat, yellowish green at maturity, margin with long cilia. Seeds ovate, with densely, short hairs, with 3-lobed, membranous arils.

Pollens isopolar, apertures 21–23. Subprolate to prolate, exine psilate, sometimes foveolate. P/E ca. 1.00–1.30.

##### Distribution.

Ceylon, India, Myanmar, the Philippines, Thailand, Vietnam, Indonesia, southwest China, Japan, and Taiwan. In Taiwan, it is mainly found in open places, on the low side of grasslands in western Taiwan and on Island Lanyu (Yang and Chen 2013).

##### Specimens examined.

**India.***Wallich 4174*, 1831 (K). **Myanmar**. *Wallich 4173*, 1831 (K). **China**. Guangdong, *S.K. Lau 1329*, s.d. (BM). Hainan, *F.C. How s.n.*, Oct. 1935 (BM). **Philippines.** Luzon, *M. Ramos & G. Edano s.n.*, Nov. 1918 (K). Mindanao, *M. Ramos & G. Edano s.n.*, Jun. 1920 (K). **Japan.** Kunigami Dist, Onna, *C. Nackejima 1810*, 3 Sep. 1967 (K). **Taiwan. Taoyuan City**: Gueishan Dist., Chienshanchiao, *T.C. Hsu 6254*, 20 Jan. 2013 (TAIF); Mt. Hutoushan, *T.C. Hsu 2439*, 1 Jan. 2010 (TAIF). **Miaoli Co.**: Zhuolan Township, *T.C. Hsu 3137*, 3 Sep. 2010 (TAIF); First Cemetery, *J.Y. Wu 59*, 25 Sep. 2021 (TNM). **Taichung City**: Hoping Dist., on the way to Mt. Tungmaoshan, *C.M. Wang 12478*, 11 Nov. 2008 (TNM); Tungshih Dist., Tungshih Forestry Farm, *C.M. Wang & K.C, Chang 1129*4, 13 Nov. 2007 (TNM). **Nantou Co.**: Yuchi Township, Lienhuachih, *S. Sasaki s.n.*, Oct. 1929 (TAI); Sun-Moon Lake, *J.Y. Wu et al. 608*, 7 Aug. 2024 (TCF). **Kaohsiung City**: Sanmin Dist., *Y.H. Tzeng 156*, 15 Aug. 2000 (TAIF). **Pingtung Co.**: Chunrih Township, Mt. Lilishan, *T.C. Hsu 4283*, 8 Jul. 2011 (TAIF). **Taitung Co.**: Lanyu Township, Yehyo Village, on the way to Mt. Hsiangaishan, *T.Y.A. Yang et al. 19003*, 5 Apr. 2007 (TNM).

#### ﻿3. Polygala japonica

Taxon classificationPlantaeFabalesPolygalaceae

Houtt., Nat. Hist. 2(10): 89. pl. 62. f. 1. 1779; Henry in List Pl. Form. 18. 1896.

097A8263-9C04-550F-B632-5D07010695FD

**Figure 9.**
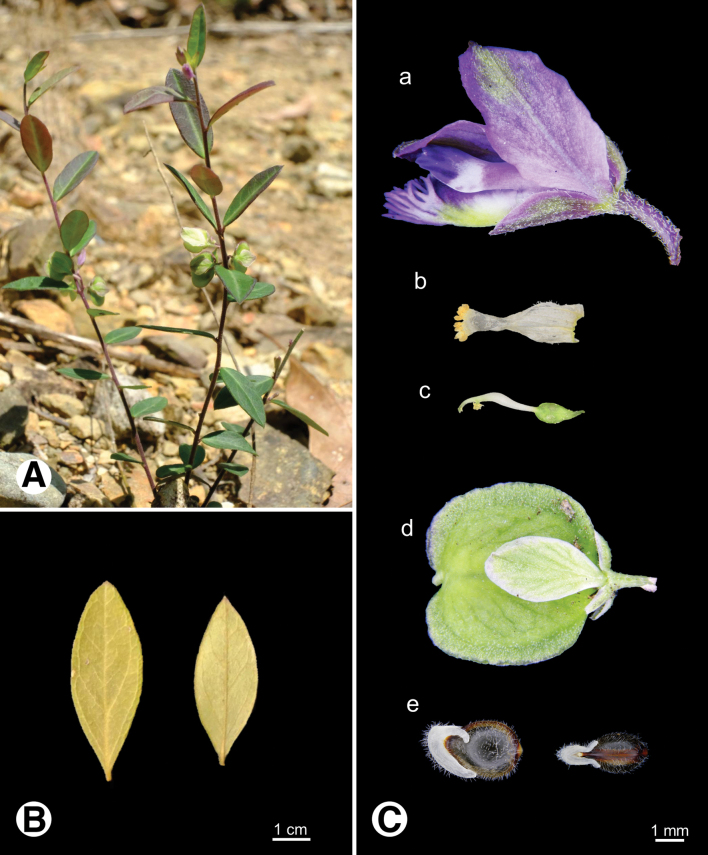
Photo of *Polygala japonica***A.** Plants in field; **B.** Leaves; **C.** Reproductive organs a flower b androecium c gynoecium d fruit e seeds.

Figs 2E, F, 9 Common name. 瓜子金

##### Type specimen.

Japan, *Thunberg s.n.*, s.d. (lectotype: G photo!).

##### Description.

Herbs, erect or ascending. Leaves simple, alternate, lanceolate, ovate, elliptic to narrowly elliptic, veins clearly visible, petioles 0.5–1 mm. Blades chartaceous. Racemes axillary. Sepals petaloid, flat, persistent. Petals purple to violet, rarely white, keel appendages violet, fringe-like, tripinnate, or more. Filaments connate at base, free at apex, anthers eight. Ovaries 2-loculed, styles simple, curved, stigmas bifid. Capsules flat, yellowish green, margin entire, purple sometimes. Seeds ovate, with sparse short hairs, arils 3-lobed, hypertrophic.

Pollens isopolar, apertures 14–19. Oblate to spheroidal, exine psilate. P/E ca. 0.71–19.

##### Distribution.

India, Myanmar, Sri Lanka, the Philippines, Moluccas, Micronesia, Russia, China, Japan, the Ryukyus, Korea, Taiwan, Papua New Guinea, Australia, and Queensland. In Taiwan, it is mainly found in open grasslands and forest edges from low to tall altitudes throughout Taiwan, Kinmen Co., Lianchlang Co., and Island Lanyu (Kerrigan 2008; Yang and Chen 2013).

##### Specimens examined.

**Philippines.** Benguet, *A. Loher 1631*, s.d. (K); Luzon, *E.D. Merrill 4368*, Oct.–Nov. 1905 (K). **Papua New Guinea.** Eastern Highlands, Obura, Kratke Mountains, *L.J. Brass & J.D. Collins 32180*, 18 Oct. 1959 (K). **Australia.** Brisbane River, *F. Mueller, s.n.*, Dec. 1856 (K). **Japan.** Yaeyama Islands, *M. Furuse 4392*, 22 Oct. 1973 (K). **Taiwan.***R. Oldham s.n.*, 1864 (BM); **Taipei City**: Beitou Dist., *Y.R. Zheng WJY362*, 29 Aug. 2023 (TCF). **New Taipei City**: Sindian Dist., Mt. Shihtoushan, *C.Y. Kuo et al. 371*, 16 May 2004 (TAIF); Tamsui Dist., *R. Oldham 76*, Apr, 1864 (BM). **Hsinchu Co.**: Guanwu, *Y.F. Huang WJY641*, 9 Sep. 2024 (TCF). **Miaoli Co.**: Taian Township, *J.Y. Wu 52*, 25 Sep. 2021 (TNM). **Taichung City**: Hoping Dist., Tahsuehshan Forest Road, Tianchi, *J.Y. Wu 172*, 8 May 2022 (TNM); *J.Y. Wu 350*, 9 Aug. 2023 (TCF). **Nantou Co.**: Renai Township, Meifeng, *J.Y. Wu 379*, 15 Oct. 2023 (TCF); Ruei-yan River, *Y.S. Huang WJY640*, 8 Sep. 2024 (TCF). **Pingtung Co.**: Hengchun Township, Fongchueisha, *J.Y. Wu et al. 500*, 3 May 2024 (TCF); Mutan Township, Hsuhai, *J.Y. Wu et al. 443*, 2 Feb. 2024 (TCF). **Hualien Co.**: Sioulin Township, Mt. Chingshuishan, *K.C. Yang & C.M. Wang 11193*, 9 May 1988 (TCF). **Taitung Co.**: Lanyu Township, Chingching Grasslands, *T.Y.A. Yang & C.F. Chen 23122*, 9 Jul. 2011 (TNM); Tianchi Lake, *J.Y. Wu 268*, 7 Feb. 2023 (TNM). **Fukien. Lianchlang Co.**: Beigan Township, Mt. Bi, *H.L. Chiang 2085*, 28 Apr. 2001 (TAIF); Nangan Township, Island Huangkuanyu, *W.H. Wu et al. 259*, 2 Apr. 2000 (TAI).

#### ﻿4. Polygala polifolia

Taxon classificationPlantaeFabalesPolygalaceae

C. Presl, Reliq. Haenk. 2: 101. 1835; Yamazaki in J. Jap. Bot. 48(5): 142. 1973.

127F3DD7-C1F9-5825-8F97-51424C499D8B

**Figure 10.**
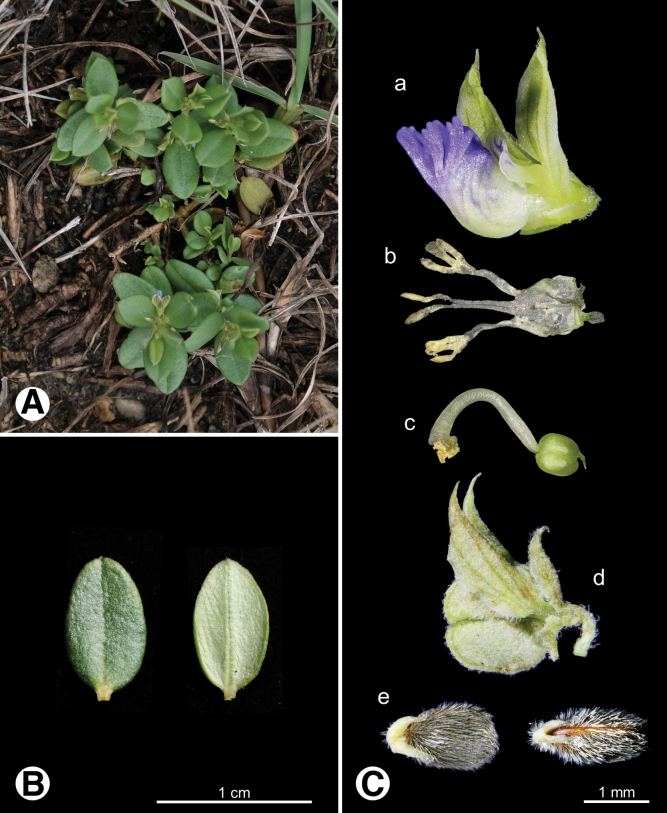
Photo of *Polygala polifolia***A.** Plants in field; **B.** Leaves; **C.** Reproductive organs a flower b androecium c gynoecium d fruit e seeds.

Figs 2G, H, 10 Common name. 無柄花瓜子金

Polygala arvensis auct. non Willd. 1802.; Yang & Liu, Manu. Taiwan Vasc. Pl. 6: 271. 2002.
Polygala glaucoides var. *hirsutula auct. non* (Arn.) Trimen., Odashima in Trop. Agr. 7: 81. 1935.
Polygala shimadai Masam., J. Soc. Trop. Agric. 3: 114, f.15. 1931. Type specimen: TAIWAN. Hsinchu Co., Hukou (Koko), *Y. Shimada 1191* (holotype: TAI!).

##### Type specimen.

Philippines, Luzon, *T. Haenke s.n.*, 1792 (holotype: MO photo!).

##### Description.

Herbs, erect or ascending. Leaves simple, alternate, ovate to elliptic, petioles 0.4–0.8 mm. Blades chartaceous. Racemes axillary. Sepals pale green, petaloid, flat, persistent. Petals three, violet, keel appendages violet, brush-like, bipinnate. Filaments connate at base and free at middle, anthers eight, two central anthers on separate filaments, lateral are sessile on two bundles of three anthers. Ovaries bilocular, ovules two, styles simple, curved, stigmas bifid slightly, glabrous. Capsules flat, yellowish green at maturity, margin entire, with long cilia. Seeds ovate, with dense hairs, arils 3-lobed, membranous.

Pollens isopolar, apertures 22–26. Oblate to spheroidal, exine patterns foveolate, reticulate. P/E ca. 0.90–1.14.

##### Distribution.

India, Bangladesh, Sri Lanka, Pakistan, Cambodia, Laos, Thailand, Vietnam, Malaysia, the Philippines, Indonesia, New Guinea, southwest China, Japan, Taiwan, and Queensland (Kerrigan 2008; Yang and Chen 2013). In Taiwan, it grows in open grasslands or wet hillsides at low altitudes in western Taiwan, Kinmen Co., and Green Island.

##### Specimens examined.

**Philippines.***Loher s.n.*, 1906 (K). Luzon, *Ramos M. s.n.*, Oct. 1909 (K). **Japan.** Ryukyu Island, *F. Miyoshi 4256* (K). **Taiwan. Taipei City**: Peitou Dist., Mt. Wuchienlienshan, *T. Suzuki 20574*, 29 Sep. 1940 (TAI). **Taoyuan City**: Bade Dist., *T. Suzuki 21374*, 23 Sep. 1941 (TAI). **Hsinchu Co.**: Hukon Township, Hukou, *Y. Simada 1191A*, 14 Sep. 1924 (TAI); Temple Lienhuashih, *T.C. Huang & W.Y. Huang 14521*, 29 Nov. 1989 (TAI). **Pingtung Co.**: Manchou Township, Nanjenshan, *T.Y.A. Yang et al. 23112*, 27 Jun. 2011 (TNM); Mutan Township, Chialoushui, *T.C. Huang & W.Y. Huang 14538*, 6 Dec. 1989 (TAI). **Fukien. Kinmen Co.**: Kinhu Township, Fengshang, *C.M. Wang & S.H. Lai 10596*, 24 Jul. 2007 (TNM); Fengshang Patrol Division Fort, *J.Y. Wu et al. 545*, 26 May 2024 (TCF).

#### ﻿ Salomonia

Taxon classificationPlantaeFabalesPolygalaceae

Lour., Flora Cochinchinensis: 14. 1790 nom. cons.

6852E3A4-0649-5EE6-A034-324AB36C839E

##### Description.

Herbs erect, annual. Leaves simple, alternate, lanceolate to wide ovate, entire, glabrous. Racemes terminal or axillary. Sepals non-petaloid, flat, persistent. Petals three, connate at base, up to half, keel appendages absent. Filaments connate to the upper end, fused with the keel, anthers four. Ovaries bilocular, heart-shape, ovules two, styles strongly curved, stigmas simple, glabrous. Capsules margin with teeth and spines. Seeds two, glabrous, arils subtle.

Pollens isopolar, oblate to spheroidal, radially symmetrical.

##### Distribution.

*Salomonia* contains 5 species distributed from tropical and subtropical Asia to Australia, with 1 species in Taiwan.

#### ﻿1. Salomonia ciliata

Taxon classificationPlantaeFabalesPolygalaceae

(L.) DC., Prodr. 1: 334. 1824; Sasaki, List Pl. Form. 256. 1928.

32B86037-C6BA-5899-A813-E37B25FC6891

**Figure 11.**
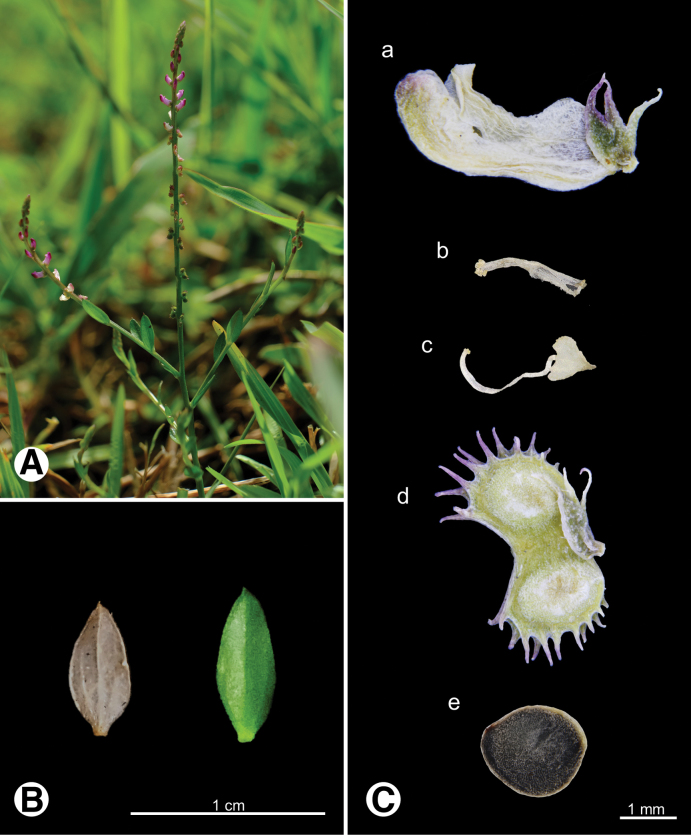
Photo of *Salomonia ciliata***A.** Plant in field; **B.** Leaves; **C.** Reproductive organs a flower b androecium c gynoecium d fruit e seed.

Figs 3A, B, 11 Common name. 齒果草

Salomonia oblongifolia DC., Prodr. 1: 334. 1824; Huang in Fl. Taiwan 3 (1^st^ edn.): 560, *pl. 782.* 1977.

##### Type specimen.

Japan, *Siebold, P.F. von, s.n.*, s.d. (lectotype: L photo!).

##### Description.

Herbs erect. Leaves simple, alternate, lanceolate to ovate, petioles 0.2–0.5 mm. Blades chartaceous. Racemes terminal or axillary. Sepals pale green, flat, persistent. Petals three, pink to pale red, keel appendages absent. Filaments connate completely, anthers four, sessile on two bundles of two anthers. Ovaries bilocular, heart-shape, ovules two, styles strongly curved, stigmas simple, glabrous. Capsules flat, green at maturity, margin dark purple, with teeth and spines. Seeds circular, glabrous, arils subtle, membranous.

Pollens isopolar, apertures 10–14. Oblate to spheroidal, exine patterns regulate and perforate. P/E ca. 0.91–0.99.

##### Distribution.

India, Nepal, Bhutan, Myanmar, Thailand, Laos, Cambodia, Vietnam, Malaysia, Papua New Guinea, the Philippines, Indonesia, China, Taiwan, Korea, Japan, and Queensland (POWO 2025). Growing in wet grasslands at low altitudes in western Taiwan.

##### Specimens examined.

**India.** Orissa, Sambalpur, Motijharan Hill, *H.F. Mooney 2890*, 3 Sep. 1947 (K). **Myanmar.** Kyaukpyu Ramree Island, *E.C. Wallace 9187*, Aug. 1945 (K). **Vietnam.***M. le Thorel 1487*, s.d. (K). **Japan.** Chiba, Hondo, *M. Furuse 44502*, 6 Sep. 1966 (K). **Taiwan. Hsinchu Co.**: Jhubei City, Lienhua Temple, *S.Y. Lu s.n.*, 27 Oct. 1989 (TAIF). **Nantou Co.**: Yuchih Township, *T.C. Hsu 9327*, 29 Jun. 2017 (TAIF); Sun Moon Lake, *J.Y. Wu 48*, 23 Aug. 2021 (TNM).

#### ﻿ Senega

Taxon classificationPlantaeFabalesPolygalaceae

(DC.) Spach, Hist. Nat. Vég. 7: 128. 1838 nom. cons.

750A1CA5-F3A9-5879-9CC2-29E962F9C84A

##### Herbs or subshrubs.

Branches thin and soft. Leaves simple, alternate, linear to laceolate, petiolate, entire, and glabrous. Racemes terminal or axillary, dense. Flowers white. Sepals five, petaloid, flat, persistent. Petals three, connate at base, keel appendages, unipinnate, semicircle or finger-like. Filaments connate at base, anthers 8. Ovaries bilocular, ovule 1 per locule, styles simple, curved in U-shape, stigmas bifid, sterile one with hairs. Capsules elliptic, pale green. Seeds 2, ellipsoidal, with membranous arils.

Pollens isopolar, subprolate, radially symmetrical.

#### ﻿1. Senega paniculata

Taxon classificationPlantaeFabalesPolygalaceae

(L.) J. F. B. Pastore & J. R. Abbott, Ann. Missouri Bot. Gard. 108: 208. 2023.

B0631DE3-463E-54B9-A30C-F9450422E2D4

**Figure 12.**
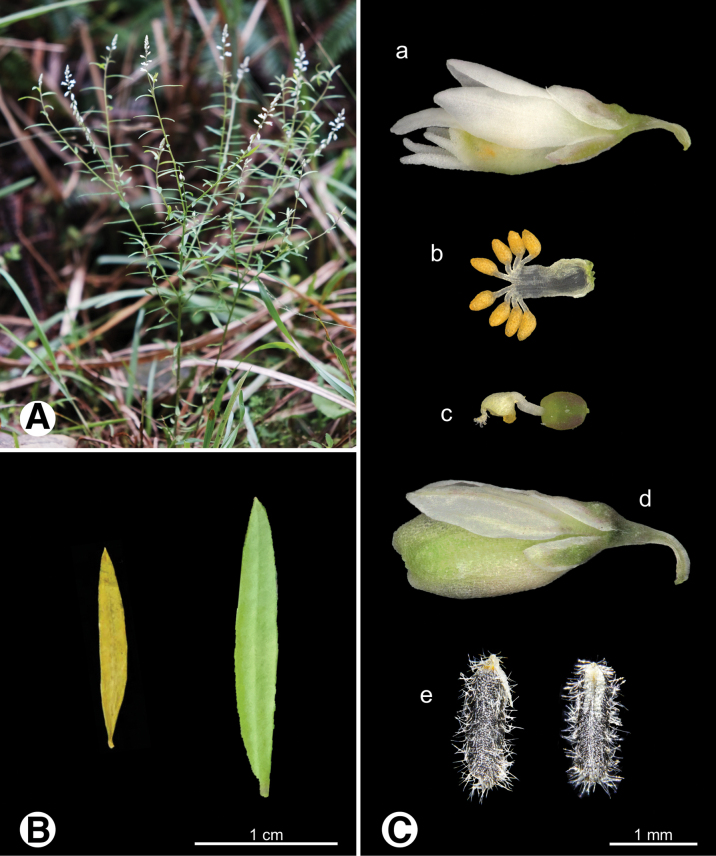
Photo of *Senega paniculata***A.** Plant in field; **B.** Leaves; **C.** Reproductive organs a flower b androecium c gynoecium d fruit e seeds.

Figs 3C, D, 12 Common name. 圓錐花遠志

##### Basionym.

*Polygala paniculata* L., Syst. Nat., ed. 10. 2: 1154. 1759; Huang in Fl. Taiwan 3 (1^st^ edn.): 558. 1977.

##### Type specimen.

Unknown, *Browne s.n.* s.d., Herb. Linn. No. 882.9 (lectotype: LINN photo!).

##### Description.

Herbs erect. Leaves simple, alternate, linear, petioles 0.5–1.2 mm. Blades herbaceous. Racemes terminal. Sepals white and pale green, petaloid, flat, persistent. Petals three, white, keel appendages white, finger-like, unipinnate. Filaments connate at base, free at upper 1/3 portion, anthers eight. Ovaries bilocular, ovule one per locule, styles simple, curved in U-shape, stigmas bifids, sterile one with hairs. Capsules elliptic, margin entire. Seeds narrowly elliptic, with sparse, short hairs, arils 2-lobed, membranous.

Pollens isopolar, apertures 8–12. Subprolate, exine psilate. P/E ca. 0.94–1.27.

##### Distribution.

Widely in the tropical and subtropical regions. Introduced and naturalized on open places and low sides at grasslands in northern and central Taiwan (Yang and Chen 2013).

##### Specimens examined.

**Brazil.***K.F.P. Martius 81*, 1841 (BM). **Colombia.***Mutis 2897*, s.d. (MA). **Paraguay.** Villa-Rica, *B. Balansa 2185*, 18 Oct. 1874 (K). **Taiwan. Taipei City**: Shihlin Dist., Mt. Taluntoushan, *P.F. Lu 18551*, 18 Jul. 2009 (TAIF). **New Taipei City**: Shiding Dist., *J.C. Wang 10989*, 13 Jul. 1999 (TAIF); Wulai Dist., Mt. Hongludishan, *J.Y. Wu et al. 558*, 1 Jun. 2024 (TCF). **Keelung City**: Anle Dist. Tawulun Battery, *C.T. Chao 2481*, 9 Sep. 2012 (TCF). **Nantou Co.**: Yuchi Township, *J.Y. Wu 47*, 23 Aug. 2021 (TNM); Lienhuachi, *C.F. Chen 3279A*, 29 Feb. 2012 (TAIF). **Ilan Co.**: Yuanshan Township, Shuanglien Pond, *S.Y. Tsai & Y.S. Liang TSY256*, 1 Apr. 2012 (TAIF). **Hualien Co.**: Xiulin Township, Tailuko (Tarokokyo), *T. Nishimura s.n.*, 9 Jul. 1929 (TAI).

## Supplementary Material

XML Treatment for Polygalaceae

XML Treatment for Epirixanthes

XML Treatment for Epirixanthes elongata

XML Treatment for Heterosamara

XML Treatment for Heterosamara arcuata

XML Treatment for Heterosamara tatarinowii

XML Treatment for Polygala

XML Treatment for Polygala arvensis

XML Treatment for Polygala chinensis

XML Treatment for Polygala japonica

XML Treatment for Polygala polifolia

XML Treatment for Salomonia

XML Treatment for Salomonia ciliata

XML Treatment for Senega

XML Treatment for Senega paniculata

## ﻿Additional information

### Use of AI

No use of AI was reported.

### Author ORCIDs

Jo-Yu Wu https://orcid.org/0009-0008-7121-7852

Hsy-Yu Tzeng https://orcid.org/0000-0003-0952-6255

T. Y. Aleck Yang https://orcid.org/0000-0002-4630-4822

### Data availability

All of the data that support the findings of this study are available in the main text or Supplementary Information.

## ﻿Appendix 1

Voucher specimens examined in this study.

***Epirixanthes elongata* Blume**

**Specimens examined. India. Assam State**: Dima Hasao dist., Haflong, N. Cadrar, *W.G. Craib s.n.*, 24 Aug. 1908 (K). **Myanmar.** Dawei Dist., Area within a radius of 12 miles from Paungdaw, half mile East of Paungdaw Power Station on east bank of Paungdaw river, *J. Keenan et al. 1527*, Sep. 1961 (K). **Malaysia. Perak State**: Gunong Kerbau, *H.C. Robinson s.n.*, s.d. (K). **Sabah State**: Imbak Canyon, *R.P. Clark et al. 22*, 16 May 2004 (K). **Thailand.** Kaosuarj, N. Sritermarut, *A.F.G. Kerr 15434*, 28 Apr. 1928 (K). **Indonesia**, Java Island, *s.coll. s.n.*, 1863 (K). **Taiwan. Pingtung Co.**: Changlo Village, *S.W. Chung et al. 7315*, 25 Aug. 2004 (TAIF); Mutan Township, *C.M. Wang et al. 16992*, s.d. (TNM), Shihzih Township, Mt. Maotzushan, *P.F. Lu 27133*, 14 Sep. 2014 (TAIF), *J.Y. Wu et al. 627*, 6 Sep. 2024 (TCF); Shouka, *C.K. Yang 533*, 24 Aug. 2004 (TNM), *T.C. Hsu 1439*, 4 Jul. 2008 (TAIF).

***Heterosamara arcuata* (Hayata) J.Y. Wu & T.Y.A. Yang**

**Specimens examined. Taiwan. Taichung City**: Hoping Dist., Mt. Pahsienshan, *J.C. Wang 11070*, 21 Jul. 1999 (TAIF), *J.Y. Wu et al. 680*, 29 Nov. 2024 (TCF); Mt. Paimaoshan, *J.Y. Wu 101*, 19 Feb. 2022 (TNM). **Nantou Co.**: Renai Township, Mt. Hsiaochushan, *F.C. Kuo & S.Y. Lu 3*, 10 Mar. 2007 (TAIF), *I.C. Kao 26*, 27 Dec. 2009 (TAIF). **Pingtung Co.**: Chunrih Township, Chinshuiying, *C.S. Kuoh 15721*, 13 May 1990 (TNM), *H.F. Yen Liu058*, 17 Nov. 1992 (TNM), *P.F. Lu 7703*, 1 Apr. 2004 (TAIF), *S.W. Chung 7012*, 1 Apr. 2004 (TAIF), *C.K. Yang 01022*, 24 Apr. 2005 (TNM), *P.F. Lu 11430*, 26 Feb. 2006 (TAIF), *C.M. Wang et al. 11544*, 30 Nov. 2007 (TNM), *P.F. Lu 20218*, 9 May 2010 (TAIF), *C.T. Lu 1772*, 21 Jan. 2011 (TAIF), *Y.C. Lu et al. 117*, 28 Jan. 2015 (TNM), *C.C. Chen Chen12*, 25 Apr. 2015 (TCF), *P.F. Lu 30298*, 27 Feb. 2017 (TAIF), *Y.C. Liu et al. YCL00561*, 11 Mar. 2017 (TNM); Tahan Forest Road, *C.M. Wang et al. 16709*, 28 Feb. 2016 (TNM), *J.Y. Wu et al. 676*, 24 Nov. 2024 (TCF); Taiwu Township, Mt. Peitawushan, *W.P. Leu 1304*, 8 Feb. 1992 (TNM), *H.F. Yen 10683*, 17 Oct. 1995 (TNM), *T.Y.A. Yang et al. 06486*, 4 Apr. 1996 (TNM), *C.S. Kuoh 13664*, 24 Jan. 1999 (TNM), *C.H. Chen et al. 3252*, 12 May 2000 (TNM), *C.K. Liou 1488*, 19 Feb. 2001 (TAIF), *S.Y. Lu s.n.*, 17 Oct. 2008 (TAIF), *S.W. Chung 13082*, 12 Nov. 2017 (TAIF), *J.Y. Shen et al. 20220816*, 16 Aug. 2022 (TNM), *Y.R. Zheng 20230115*, 15 Jan. 2023 (TNM); Wutai Hsiang, *H.F. Yen 03573*, 1 Jan. 1990 (TNM), *H.F. Yen 06018*, 31 Mar. 1992 (TNM), *C.H. Chen et al. 511*, 1 Apr. 1994 (TNM), *C.M. Wang et al. 09400*, 9 Oct. 2006 (TNM), Mt. Sung, *S.Y. Lu 22832*, 12 May 1988 (TAIF). **Taitung Co.**: Jinfong Township, Mt. Talilishan, *T.C. Hsu 6911*, 1 Mar. 2014 (TAIF).

***Heterosamara tatarinowii* (Regel) Paiva**

**Specimens examined. India.** Himachal Pradesh State, Chil Forest above Waterfalls Simla, *J.S. Gamble 5091A*, 16 Sep. (K). Lachooni, *J.D.H. s.n.*, 15 Aug. (K). **Bhutan.** Thimphu Dist., Dechhenphu N of Thimphu, *I.W.J. Sinclair & D.G. Long 4817*, 5 Sep. 1984 (K). **China.** Yunnan Dist., *J. Cavalerie 4394*, 1917 (K); Central Yunnan, between Likiang and Talifu, *J.F. Rock 6288*, 13 Sep. 1922 (K). **Japan.** Hondo, Nippara in Musashi, *M. Togasi 1223*, 14 Sep. 1955 (K). Mrioka, *P. Faurie 618[3*], 28 Aug. 1890 (K). **Taiwan. Hsinchu Co.**: Jianshih Township, Taikang, *P.F. Lu 25953*, 10 Aug. 2013 (TAIF). **Nantou Co.**: Jhushan Township, *M.J. Jung 1733*, 21 Oct. 2007 (TAIF); Renai Township, *W.C. Leong 1308*, 7 Aug. 1999 (TNM), *J.Y. Wu 212*, 10 Aug. 2022 (TNM); Jihhsinkang- Hohuan tunnel, *C.M. Wang & C.P. Lu W05145*, 7 Aug. 2001 (TNM); Lushan, *H.F. Yen 25069*, 3 Oct. 2002 (TNM); Tunyuan, *C.K. Yang 00001*, 3 Sep. 2001 (TNM), *T.Y.A. Yang 11119*, 2 Sep. 1998 (TNM), *T.Y.A. Yang et al. 19771*, 17 Sep. 2007 (TNM); Yunhai, *C.H. Chen et al. 2434*, 31 Jul. 1998 (TNM), *C.H. Chen et al. 03428*, 6 Sep. 2000 (TNM); Nengkao Cross-Ridge Historic Trail, *P.F. Lu 18659*, 1 Aug. 2009 (TAIF), *C.T. Chao et al. 3110*, 6 Aug. 2013 (TCF); Sinyi Township, Divine Tree, *H.F. Yen 01148*, 28 Jun. 1987 (TNM); Fenglin Suspension Bridge, *P.F. Lu 26007*, 28 Sep. 2013 (TAIF); Futzu Cleef, *K.C. Huang 438*, 20 Aug. 2008 (TNM); Pagou Lass, *P.F. Lu 22781*, 3 Sep. 2011 (TAIF); Shang Tungpu to Lolo Sanwu, *T.Y.A. Yang 3465*, 31 Jul. 1987 (TNM). **Chiayi Co.**: Alishan Township, *T.Y.A. Yang et al. 19720*, 16 Sep. 2007 (TNM); Alishan Highway, *P.F. Lu 22657*, 13 Aug. 2011 (TAIF), *S.W. Chung 10464*, 17 Sep. 2011 (TAIF), *C.T. Chao 2512*, 11 Sep. 2012 (TCF), *C.M. Wang et al. 17150*, 12 Sep. 2017 (TNM); Nanhsi Logging Trail, *P.H. Lee 3465*, 15 Aug. 2005 (TAIF); Fanlu Township, Lungtou, *C.M. Wang & C.Y. Li W05123*, 28 Jul. 2001 (TNM); Jhuci Township, Shihcho, *T.C. Hsu 4651*, 15 Sep. 2011 (TAIF); Meishan Township, Meishan, *C.M. Wang 00335*, 9 Sep. 1993 (TNM). **Kaohsiung City**: Taoyuan Dist., Tianchi, *C.S. Kuoh 15022*, 14 Sep. 1999 (TNM); Bridge Likuangchiao, *T.Y.A. Yang et al. 7488*, 13 Sep. 1996 (TNM). **Ilan Co.**: Datong Township, Suchi-Nanshan, *C.M. Wang s.n.*, 6 Aug. 1992 (TNM). **Hualien Co.**: Juisui Township, Juisui Forest Road 29.5–32 K, *C.M. Wang et al. 11359*, 18 Nov. 2007 (TNM); Shoufong Township, *S. Saito 425*, 23 Apr. 1927 (TNM); National Dong-Hwa University, *T.T. Chen 11214*, 10 Apr. 2001 (TAIF), *W.Y. Wang 2165*, 17 Jan. 2016 (TAIF), *C.T. Chao Chao4750*, 18 Feb. 2019 (TAIF); Sioulin Township, Hoping Logging Trail, *H.W. Lin 1236*, 13 Aug. 1999 (TAIF), *S.W. Chung & T.C. Hsu 8115*, 3 Sep. 2004 (TAIF); Kuanyuan Lodge, *Liu Liu30*, 25 Jul. 1994 (TNM); Tienchih, *P.F. Lu 29811*, 21 Aug. 2016 (TAIF); Tzuen, *P.F. Lu 27083*, 24 Aug. 2014 (TAIF). **Taitung Co.**: Haiduan Township, Tienlung Historical Trail, *P.F. Lu 10563*, 16 Oct. 2005 (TAIF).

***Polygala arvensis* Willd.**

**Specimens examined. India.** Goalpara, *s.coll. 4172*, s.d. (K). Murree-Kohala Road, *R.R. Stewart 5068*, 30 Aug. 1919 (K). **Taiwan. Tainan City**: Guanmiao Dist., *T.H. Hsieh s.n.*, 11 Oct. 2020 (TNM); Mujiashan, *C.Y. Chang et al. 1063*, 9 Jan. 2016 (TNM); Xinhua Dist., Mujiashan Grasslands, *J.Y. Wu 63*, 2 Oct. 2021 (TNM).

***Polygala chinensis* L.**

**Specimens examined. India.***Wallich 4174*, 1831 (K). **Myanmar**. *Wallich 4173*, 1831 (K). **China**. Guangdong, *S.K. Lau 1329*, s.d. (BM). Hainan, *F.C. How s.n.*, Oct. 1935 (BM). **Philippines.** Luzon, *M. Ramos & G. Edano s.n.*, Nov. 1918 (K). Mindanao, *M. Ramos & G. Edano s.n.*, Jun. 1920 (K). **Japan.** Kunigami Dist, Onna, *C. Nackejima 1810*, 3 Sep. 1967 (K). **Taiwan. Taoyuan City**: Gueishan Dist., Chienshanchiao, *T.C. Hsu 6254*, 20 Jan. 2013 (TAIF); Mt. Hutoushan, *T.C. Hsu 2439*, 1 Jan. 2010 (TAIF). **Miaoli Co.**: Zhuolan Township, *T.C. Hsu 3137*, 3 Sep. 2010 (TAIF); First Cemetery, *C.T. Chao 1470*, 25 Sep. 2010 (TCF), *J.Y. Wu 59*, 25 Sep. 2021 (TNM). **Taichung City**: Hoping Dist., on the way of Mt. Tungmaoshan, *C.M. Wang 12478*, 11 Nov. 2008 (TNM); Nantun Dist., Wangkaoliao, *C.M. Wang et al.10851*, 16 Oct. 2007 (TNM); Tungshih Dist., Tungshih Forestry Farm, *C.M. Wang & K.C, Chang 1129*4, 13 Nov. 2007 (TNM). **Nantou Co.**: Yuchi Township, Lienhuachih, *S. Sasaki s.n.*, Oct. 1929 (TAI); Sun-Moon Lake, *C.M. Wang & S.K. Yu 11214*, 8 Nov. 2007 (TNM), *C.H. Chen & C.M. Wang 8516*, 28 Nov. 2007 (TNM), *J.Y. Wu et al. 608*, 7 Aug. 2024 (TCF), *J.Y. Wu et al. 652*, 26 Oct. 2024 (TCF). **Kaohsiung City**: Sanmin Dist., *Y.H. Tzeng 156*, 15 Aug. 2000 (TAIF). **Pingtung Co.**: Chunrih Township, Mt. Lilishan, *T.C. Hsu 4283*, 8 Jul. 2011 (TAIF). **Taitung Co.**: Lanyu Township, Yehyo Village, on the way of Mt. Hsiangaishan, *T.Y.A. Yang et al. 19003*, 5 Apr. 2007 (TNM).

***Polygala japonica* Houtt.**

**Specimens examined. Philippines.** Benguet, *A. Loher 1631*, s.d. (K); Luzon, *E.D. Merrill 4368*, Oct.–Nov. 1905 (K). **Papua New Guinea.** Eastern Highlands, Obura, Kratke Mountains, *L.J. Brass & J.D. Collins 32180*, 18 Oct. 1959 (K). **Australia.** Brisbane River, *F. Mueller, s.n.*, Dec. 1856 (K). **Japan.** Yaeyama Islands, *M. Furuse 4392*, 22 Oct. 1973 (K). **Taiwan.***R. Oldham s.n.*, 1864 (BM); **Taipei City**: Beitou Dist., *Y.R. Zheng WJY362*, 29 Aug. 2023 (TCF). **New Taipei City**: Sindian Dist., Mt. Shihtoushan, *C.Y. Kuo et al. 371*, 16 May 2004 (TAIF); Tamsui Dist., *R. Oldham 76*, Apr, 1864 (BM); Wulai Dist., Wulai, *S.W. Chung 9127*, 9 Jun. 2008 (TAIF). **Hsinchu Co.**: Guanwu, *Y.F. Huang WJY641*, 9 Sep. 2024 (TCF). **Miaoli Co.**: Taian Township, *J.Y. Wu 52*, 25 Sep. 2021 (TNM). **Taichung City**: Hoping Dist., Mt. Hsuehshan, *S.M. Kuo 331*, 10 Jul. 2001 (TAIF), *Y.C. Lin et al. 27*, 15 Aug. 2020 (TCF); Tahsuehshan Forest Road, Tianchi, *J.Y. Wu 172*, 8 May 2022 (TNM); Tahsuehshan Forest Road 50K, *J.Y. Wu 350*, 9 Aug. 2023 (TCF). **Nantou Co.**: Renai Township, Meifeng, *J.Y. Wu 379*, 15 Oct. 2023 (TCF); Provincial Highway 14, Xiaofengkou, *H.P. Hsieh 25*, 4 May 2021 (TCF); Ruei-yan River, *Y.S. Huang WJY640*, 8 Sep. 2024 (TCF). **Pingtung Co.**: Hengchun Township, Fongchueisha, *J.Y.Wu et al. 500*, 3 May 2024 (TCF); Mt. Santaishan, *T.C. Hsu 5273*, 17 Jan. 2012 (TAIF); Mutan Township, Hsuhai, *J.Y. Wu et al. 443*, 2 Feb. 2024 (TCF); Manchou Township, daifong grassland, *J.Y. Wu et al. 696*, 7 Feb. 2025 (TCF). **Hualien Co.**: Sioulin Township, Mt. Chingshuishan, *K.C. Yang & C.M. Wang 11193*, 9 May 1988 (TCF); Chuilu Ancient Trail, *C.F. Chen 3622*, 6 Jun. 2012 (TAIF). **Taitung Co.**: Lanyu Township, Chingching Grasslands, *T.Y.A. Yang & C.F. Chen 23122*, 9 Jul. 2011 (TNM); Tianchi Lake, *J.Y. Wu 268*, 7 Feb. 2023 (TNM). **Fukien. Lianchlang Co.**: Beigan Township, Mt. Bi, *H.L. Chiang 2085*, 28 Apr. 2001 (TAIF); Nangan Township, Island Huangkuanyu, *W.H. Wu et al. 259*, 2 Apr. 2000 (TAI).

***Polygala polifolia* C.Presl**

**Specimens examined. Japan.** Ryukyu Island, *F. Miyoshi 4256* (K). **Philippines.***Loher s.n.*, 1906 (K). Luzon, *Ramos M. s.n.*, Oct. 1909 (K), Fénix E. s.n., Nov. 1917 (K). **Taiwan. Taipei City**: Peitou Dist., Mt. Wuchienlienshan, *T. Suzuki 20574*, 29 Sep. 1940 (TAI). **Taoyuan City**: Bade Dist., *T. Suzuki 21374*, 23 Sep. 1941 (TAI). **Hsinchu Co.**: Hukon Township, Hukou, *Y. Simada 1191A*, 14 Sep. 1924 (TAI); Temple Lienhuashih, *T.C. Huang & W.Y. Huang 14521*, 29 Nov. 1989 (TAI). **Pingtung Co.**: Manchou Township, Nanjenshan, *T.Y.A. Yang et al. 23112*, 27 Jun. 2011 (TNM); Mutan Township, Chialoushui, *T.C. Huang & W.Y. Huang 14538*, 6 Dec. 1989 (TAI). **Fukien. Kinmen Co.**: Kinhu Township, Fengshang, *C.M. Wang & S.H. Lai 10596*, 24 Jul. 2007 (TNM); Fengshang Patrol Division Fort, *J.Y. Wu et al. 545*, 26 May 2024 (TCF).

***Salomonia ciliata* DC.**

**Specimens examined. India.** Orissa, Sambalpur, Motijharan Hill, *H.F. Mooney 2890*, 3 Sep. 1947 (K). **Myanmar.** Kyaukpyu Ramree Island, *E.C. Wallace 9187*, Aug. 1945 (K). **Vietnam.***M. le Thorel 1487*, s.d. (K). **Japan.** Chiba, Hondo, *M. Furuse 44502*, 6 Sep. 1966 (K). **Taiwan. Hsinchu Co.**: Jhubei City, Lienhua Temple, *S.Y. Lu s.n.*, 27 Oct. 1989 (TAIF). **Nantou Co.**: Yuchih Township, *T.C. Hsu 9327*, 29 Jun. 2017 (TAIF); Shuishe, *C.M. Wang & P.F. Lu 16951*, 26 Aug. 2016 (TNM); Sun Moon Lake, *S.K. Yu 63*, 15 Aug. 2007 (TNM), *C.M. Wang & S.C. Hsiao 12299*, 1 Sep. 2008 (TNM), *C.T. Chao et al. 3474*, 22 Aug. 2014 (TCF), *C.Y. Chang et al. 1361*, 26 Aug. 2016 (TNM), *T.C. Li 136*, 26 Aug. 2016 (TCF), *J.Y. Wu 48*, 23 Aug. 2021 (TNM).

***Senega paniculata* (L.) J. F. B. Pastore & J. R. Abbott**

**Specimens examined. Brazil.***K.F.P. Martius 81*, 1841 (BM). **Colombia.***Mutis 2897*, s.d. (MA). **Paraguay.** Villa-Rica, *B. Balansa 2185*, 18 Oct. 1874 (K). **Taiwan. Taipei City**: Shihlin Dist., Mt. Taluntoushan, *P.F. Lu 18551*, 18 Jul. 2009 (TAIF). **New Taipei City**: Shiding Dist., *J.C. Wang 10989*, 13 Jul. 1999 (TAIF); Wulai Dist., Mt. Hongludishan, *J.Y. Wu et al. 558*, 1 Jun. 2024 (TCF); Wulai, *S.Y. Lu 15792*, 19 Apr. 1985 (TAIF). **Keelung City**: Anle Dist. Tawulun Battery, *C.T. Chao 2481*, 9 Sep. 2012 (TCF). **Nantou Co.**: Yuchi Township, *J.Y. Wu 47*, 23 Aug. 2021 (TNM); Lienhuachi, *C.F. Chen 3279A*, 29 Feb. 2012 (TAIF). **Ilan Co.**: Yuanshan Township, Shuanglien Pond, *S.Y. Tsai & Y.S. Lianf TSY256*, 1 Apr. 2012 (TAIF). **Hualien Co.**: Xiulin Township, Tailuko (Tarokokyo), *T. Nishimura s.n.*, 9 Jul. 1929 (TAI).

## ﻿Appendix 2

**Table A1.** Specimens used in this study.

Species	Collector and coll. no.	date	herbarium code	Applications
Epirixanthes elongata	*C.M. Wang et al. 16992*	s.d.	TNM	Morph., palyn.
-	*P.F. Lu 27133*	14 Sep. 2014	TAIF	Morph., palyn.
-	*C.K. Yang 533*	24 Aug. 2004	TNM	Morph., palyn
-	*J.Y. Wu et al. 627*	6 Sep. 2024	TCF	Morph., palyn., photo.
Heterosamara tatarinowii	*J.Y. Wu 212*	10 Aug. 2022	TNM	Morph., palyn., photo.
-	*T.Y.A. Yang et al. 19720*	16 Sep. 2007	TNM	Morph., palyn.
-	*T.Y.A. Yang et al. 19771*	17 Sep. 2007	TNM	Morph., palyn.
Polygala arcuata	*G. Nakahara s.n.*,	Feb. 1907	TAIF (Isotype)	Morph.
-	*J.Y. Shen et al. 20220816*	16 Aug. 2022	TNM	Morph., palyn., photo.
-	*J.Y. Wu 101*	19 Feb. 2022	TNM	Morph., palyn., photo.
-	*J.Y. Wu et al. 676*	24 Nov. 2024	TCF	Morph., photo.
-	*J.Y. Wu et al. 680*	29 Nov. 2024	TCF	Morph.
-	*Y.R. Zheng 20230115*	15 Jan. 2023	TNM	Morph., palyn.
P. arvensis	*C.Y. Chang et al. 1063*	9 Jan. 2016	TNM	Morph., palyn.
-	*J.Y. Wu 63*	2 Oct. 2021	TNM	Morph., palyn., photo.
-	*T.H. Hsieh s.n.*	11 Oct. 2020	TNM	Morph., palyn., photo.
P. chinensis	*J.Y. Wu 59*	25 Sep. 2021	TNM	Morph., palyn., photo.
-	*J.Y. Wu et al. 608*	7 Aug. 2024	TCF	Morph., palyn., photo.
-	*J.Y. Wu et al. 652*	26 Oct. 2024	TCF	Morph., palyn.
P. japonica	*J.Y. Wu 52*	25 Sep. 2021	TNM	Morph., palyn.
-	*J.Y. Wu 172*	8 May 2022	TNM	Morph., palyn., photo.
-	*J.Y. Wu 350*	9 Aug. 2023	TCF	Morph., palyn.
-	*J.Y.Wu et al. 500*	3 May 2024	TCF	Morph.
P. polifolia	*T.Y.A. Yang et al. 23112*	27 Jun. 2011	TNM	Morph., palyn.
-	*J.Y. Wu 142*	Aug. 2021	TNM	Morph., palyn., photo.
-	*J.Y. Wu et al. 545*	26 May 2024	TCF	Morph., palyn., photo.
Salomonia ciliata	*C.M. Wang & P.F. Lu 16951*	26 Aug. 2016	TNM	Morph., palyn.
-	*C.Y. Chang et al. 1361*	26 Aug. 2016	TNM	Morph., palyn.
	*J.Y. Wu 48*	23 Aug. 2021	TNM	Morph., palyn., photo.
Senega paniculata	*C.F. Chen 3279A*	29 Feb. 2012	TAIF	Morph.
-	*J.Y. Wu 47*	23 Aug. 2021	TNM	Morph., palyn., photo.
-	*J.Y. Wu et al. 558*	1 Jun. 2024	TCF	Morph., palyn., photo.

Morph.: morphological, palyn.: palynological, photo.: photography.

## References

[B1] Abbott JR (2009) Revision of *Badiera* (Polygalaceae) and phylogeny of the Polygaleae. Dissertation, University of Florida, Gainesville, Florida, U.S.A. https://ufdc.ufl.edu/UFE0041138/00001/pdf

[B2] Banks H Bente BK Forest F Crane PR (2008) Pollen morphology of the family Polygalaceae (Fabales). Botanical Journal of the Linnean Society 156: 253–289. 10.1111/j.1095-8339.2007.00723.x

[B3] Bashyal S Joshi GP Pant DR (2025) *Polygala arvensis* Willd.: A taxonomic note for the Flora of Nepal. Botanica Orientalis. Journal of Plant Sciences 16(1): 43–45. 10.3126/botor.v16i1.79987

[B4] Chen SK Ma H-Y Parnell JAN (2008) Polygalaceae. In: Wu et al. (Eds) Flora of China, Vol. 11. Science Press, Beijing and St. Louis, 139–159.

[B5] Chung SW (2017) Illustrated flora of Taiwan 4. Owl Publishing House, Cité Publishing Ltd. Taipei, Taiwan, 275–279. [in Chinese]

[B6] Dančák M Hroneš M Sukri RS Metali F Joffre AA (2017) Novitates Bruneienses, 9. A synopsis of *Epirixanthes* (Polygalaceae) in Brunei Darussalam and notes on species elsewhere. Gardens’ Bulletin (Singapore) 69: 179–187. 10.26492/gbs69(2).2017-03

[B7] Erdtman G (1960) The acetolysis method. A revised description. Svensk Botanisk Tidskrift 54: 561–564.

[B8] Eriksen B Persson C (2007) Polygalaceae. In: Kubitzki K (Ed.) The Families and Genera of Vascular Plants 9.Springer, Berlin, 345–363. 10.1007/978-3-540-32219-1_41

[B9] Hsieh C-F Hsieh T-H Lai I-L (1995) *Epirixanthes elongata* Bl.- a New Record to the Flora of Taiwan. Taiwania 40(4): 381–384. 10.6165/tai.1995.40.381

[B10] Huang TC (1977) Polygalaceae. In: Huang et al. (Eds) Flora of Taiwan 3 (1^st^ edn.). Editorial Committee of Flora of Taiwan, Department of Botany, National Taiwan University, Taipei, 557–562.

[B11] Huang TC (1993) Polygalaceae. In: Huang et al. (Eds) Flora of Taiwan 3 (2^nd^ edn.). Editorial Committee of Flora of Taiwan, Department of Botany, National Taiwan University, Taipei, 571–578.

[B12] Huang TC (2003) Polygalaceae. In: Huang et al. (Eds) Flora of Taiwan 6 (2^nd^ edn.). Editorial Committee of Flora of Taiwan, Department of Botany, National Taiwan University, Taipei, 72.

[B13] Kerrigan R (2008) A taxonomic revision of *Polygala* L. in northern Australia. Masters (Research) thesis, James Cook University, 290 pp. 10.25903/ecqv-fk90

[B14] Lacaille-Dubois MA Delaude C Mitaine-Offer AC (2020) A review on the phytopharmacological studies of *Polygala.* Journal of Ethnopharmacology 249(12): 112417. 10.1016/j.jep.2019.112417 31765761

[B15] Mongalo NI McGaw LJ Finnie JF van Staden J (2015) *Securidaca longipedunculata* Fresen (Polygalaceae): A review of its ethnomedicinal uses, phytochemistry, pharmacological properties and toxicology. Journal of Ethnopharmacology 165(13): 215–226. 10.1016/j.jep.2015.02.041 25724970

[B16] Paiva JAR (1998) Polygalarum africanarum et madagascariensium prodromus atque gerontogaei generis *Heterosamara* Kuntze, a genere *Polygala* L. segregate et a nobis denuo recepti, synopsis monographica. In: Casas FJF (Ed.) Fontqueria 50, Madrid, 1–346.

[B17] Pastore JFB Abbott JR Neubig KM Whitten WM Mascarenhas RB Mota MCA van den Berg C (2017) A molecular phylogeny and taxonomic notes in *Caamembeca* (Polygalaceae). Systematic Biology 42(1): 54–62. 10.1600/036364417X694935

[B18] Pastore JFB Abbott JR Neubig KM Berg CBD Mota MCDA Cabral A Whitten WM (2019) Phylogeny and biogeography of *Polygala* (Polygalaceae). Taxon 68(12): 1–19. 10.1002/tax.12119

[B19] Pastore JF Martinez A Abbott J Neubig K (2023) Toward New Generic Delimitations in Polygalaceae II: *Senega.* Annals of the Missouri Botanical Garden 108: 126–249. 10.3417/2023754

[B20] Pendry CA (2010) *Epirixanthes compressa* Pendry, a new mycoheterotrophic species of Polygalaceae from Thailand. Thai Forest Bulletin. Botany. Bangkok 38: 184–186. https://li01.tci-thaijo.org/index.php/ThaiForestBulletin/article/view/24433

[B21] POWO (2025) Plants of the World Online. Facilitated by the Royal Botanic Gardens, Kew. powo.science.kew.org [accessed on 25 Jul. 2025]

[B22] Chase MW Christenhusz MJM Fay MF Byng JW Judd WS Soltis DE Mabberley DJ Sennikov AN Soltis PS Stevens PF (2016) An update of the angiosperm phylogeny group classification for the orders and families of flowering plants: APG IV. Botanical Journal of the Linnean Society 181: 1–20. 10.1111/boj.12385

[B23] Tsukaya H Suleiman M Okada H (2016) A new species of *Epirixanthes* (Polygalaceaea) from Imbak Canyon, Sabah, Borneo. Phytotaxa 266: 146–150. 10.11646/phytotaxa.266.2.9

[B24] Van der Meijden R (1988) Polygalaceae. In: van Steenis de Wilde (Eds) Flora Malesiana, ser.I, Vol. 10(1). Kluwer Academic Publishers, Dordrecht, Boston and London, 455–539.

[B25] Yang TYA Chen CF (2013) A revision of *Polygala* L. (Polygalaceae) in Taiwan. Taiwania 58(3): 156–162. 10.6165/tai.2013.58.156

